# *De Novo* Production of Xanthohumol by a Metabolically Engineered *Escherichia coli*

**DOI:** 10.1021/acssynbio.5c00221

**Published:** 2025-09-25

**Authors:** Daniela Gomes, Joana Santos, Armando Venâncio, Joana L. Rodrigues, Nigel S. Scrutton, Ligia R. Rodrigues

**Affiliations:** † CEB-Centre of Biological Engineering, 56059 Universidade do Minho, Campus de Gualtar, 4710-057 Braga, Portugal; ‡ LABBELS Associate Laboratory, Braga 4710-057, Portugal; § Manchester Institute of Biotechnology, 5292 The University of Manchester, 131 Princess Street, Manchester M1 7DN, U.K.

**Keywords:** xanthohumol, Escherichia coli, metabolic engineering, synthetic biology, heterologous production, CRISPR-Cas12a

## Abstract

Xanthohumol is a prenylflavonoid from hops with relevant bioactivities. Microbial production has emerged as a sustainable and potentially economic solution to produce it. Herein, we constructed a pathway for the *de novo* production of xanthohumol in *Escherichia coli*. Since the xanthohumol pathway depends on the availability of dimethylallyl pyrophosphate (DMAPP) and *S-*adenosylmethionine (SAM), SAM synthase (*metK*) was integrated into the genome of *E. coli* strains with previously engineered DMAPP pathways. Eleven prenyltransferases (PT) and the *O*-methyltransferase (OMT) from *Humulus lupulus* (*Hl*OMT1) were tested. *E. coli* M-PAR-121:*Bl*IDI:*metK*, constructed by integrating *metK* into the *E. coli* strain with integration of isopentenyl diphosphate isomerase (IDI) from *Bacillus licheniformis* (*E. coli* M-PAR-121:*Bl*IDI) and expressing CdpC3PT from *Neosartorya fischeri* and *Hl*OMT1 in combination with the naringenin chalcone pathway, was the best producer. This strain was able to produce 7.3 mg/L of desmethylxanthohumol and 5.3 mg/L of xanthohumol in the bioreactor, representing the first report of *de novo* production of xanthohumol in *E. coli*.

## Graphical abstract

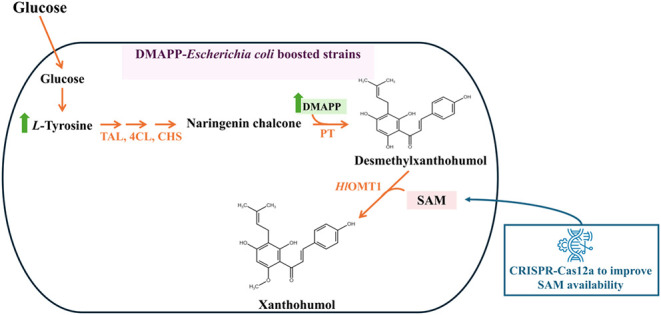

## 1 Introduction

Xanthohumol belongs to the prenylflavonoids class, and it is naturally produced in *Humulus lupulus*, commonly known as hops. Hops are one of the main ingredients used in the production of beer.^1,2^ Xanthohumol is considered a powerful compound with several reported health benefits for human health, namely as an anticancer, antioxidant, anti-inflammatory, and antiviral agent.^3–12^ For example, this compound is reported to exert effects on several types of cancer by inducing apoptosis and inhibiting cell proliferation^3,10^ Despite the potential of this compound, xanthohumol content in dry hop matter depends on the hop’s variety, ranging from 0.1% to 1%.^13,14^ For example, Stevens et al. reported a xanthohumol content of only 0.48% in dry hop matter.^15^ As a result, extracting xanthohumol from hops in amounts for incorporation in pharmaceutical and nutraceutical compounds is difficult. Its production is also dependent on climatic and seasonal variations.^13^

The use of microorganisms for the synthesis of plant natural compounds has emerged as a sustainable and economically friendly way to obtain them.^16^ Understanding the biosynthetic steps involved in the synthesis of xanthohumol in hops is essential to construct a microbial cell factory able to produce it. In recent years, all steps of the biosynthetic pathway in the original plants were uncovered, facilitating the construction of a heterologous pathway. The biosynthetic pathway to naringenin chalcone has been extensively studied toward the heterologous production of naringenin and other derived compounds in microorganisms.^17–22^ However, the final two steps leading to xanthohumol production have been less explored.^23,24^

The xanthohumol biosynthetic pathway can be constructed by first assembling a naringenin chalcone biosynthetic pathway (Figure 1). Then, naringenin chalcone is prenylated by a prenyltransferase (PT) that uses dimethylallyl diphosphate (DMAPP) as an extended substrate, producing desmethylxanthohumol. Afterward, desmethylxanthohumol is converted into xanthohumol by an *O-*methyltransferase (OMT), using *S-*adenosylmethionine (SAM) as a methyl donor. Both DMAPP and SAM are naturally produced in microorganisms. However, the intracellular concentration of these compounds can be limiting since they are involved in key metabolic reactions.^25–27^

**Figure 1:**
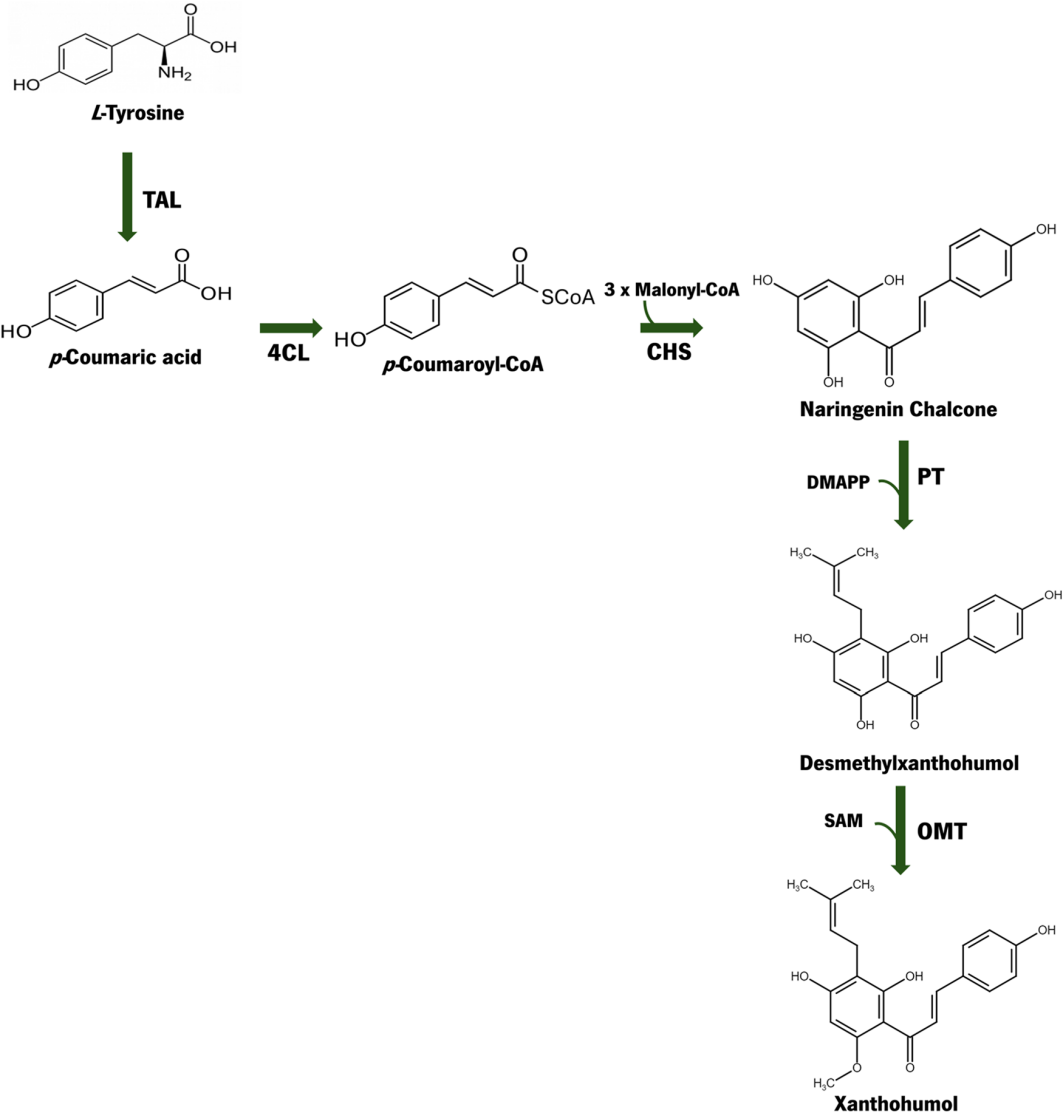
Xanthohumol biosynthetic pathway. The pathway is composed of tyrosine-ammonia lyase (TAL), 4-coumarate:CoA ligase (4CL), chalcone synthase (CHS), prenyltransferase (PT), and *O*-methyltransferase (OMT). Malonyl-CoA, dimethylallyl diphosphate (DMAPP), and S-adenosylmethionine (SAM) are required by CHS, PT, and OMT, respectively.

To the best of our knowledge, only *Saccharomyces cerevisiae* has previously been engineered to produce xanthohumol.^28^ Several optimizations of the metabolic flux of intermediaries and extender substrates of the pathway were performed to achieve the final production of desmethylxanthohumol and xanthohumol. A knockout of the phenylpyruvate decarboxylase gene and overexpression of feedback-inhibition-resistant versions of 3-deoxy-d-arabino-heptulosonic acid 7-phosphate synthase and chorismate mutase were performed to improve l-tyrosine biosynthesis. DMAPP availability was also improved by overexpressing a truncated variant of the 3-hydroxy-3-methyl-glutaryl-CoA reductase and isopentenyl diphosphate isomerase (IDI), the rate-limiting steps of the mevalonate pathway. Moreover, a mutation of the gene encoding farnesyl diphosphate (FPP) synthase was performed to decrease the rate of DMAPP conversion into FPP. PT1 from *H. lupulus* (*Hl*PT1), identified as the enzyme responsible for the prenylation step in hops, was modified to improve its catalytic activity through the truncation of the N-terminal signal peptide. A codon-optimized version of OMT1 from *H. lupulus* (*Hl*OMT1) was also overexpressed to convert desmethylxanthohumol into xanthohumol. With all of these optimizations, 2.2 mg/L of desmethylxanthohumol and 0.14 mg/L of xanthohumol from glucose were produced in shake flask experiments.^28^

Since *Escherichia coli* was extensively exploited as a chassis for the heterologous production of flavonoids, this microorganism could also be interesting for constructing the xanthohumol pathway.^17,18,20,21,29^ Compared to *S. cerevisiae*, *E. coli* has higher growth rates and a shorter doubling time.^30^ Additionally, the expression levels of heterologous genes in *E. coli* are usually higher than in *S. cerevisiae*.^31^ These features are essential for the development of an efficient and competitive production process.^30^ Considering that in this work, we aimed to achieve *de novo* production of xanthohumol in *E. coli* for the first time. *E. coli* M-PAR-121 strain with the integration of IDI from *Bacillus licheniformis* (*Bl*IDI) and of the native SAM synthase (*metK*) of the genome (*E. coli* M-PAR-121:*Bl*IDI:*metK*) expressing the naringenin chalcone pathway, CdpC3PT from *Neosartorya fischeri* *, and Hl*OMT1 was selected as the best producing strain, being able to produce 21.5 μM (7.3 mg/L) of desmethylxanthohumol and 14.8 μM (5.3 mg/L) of xanthohumol at a bioreactor scale. As far as we know, this work corresponds to the first report of the *de novo* production of xanthohumol in *E. coli* and the highest production reported in any host.

## 2 Results and Discussion

### 2.1 Assembly of PT and OMT1 Steps and Evaluation of *De Novo* Production of Xanthohumol in *E. coli* M-PAR-121

The functional expression of a PT and an OMT is required to efficiently convert naringenin chalcone into xanthohumol (Figure 1). PT converts naringenin chalcone into the intermediary desmethylxanthohumol. For this step, three PTs from plants and eight PTs from microbial sources were tested. Codon-optimized versions of PT from *H. lupulus* (*Hl*PT1), PT3 from *Cannabis sativa* (*Cs*PT3), N8DT-1 from *Sophora flavescens* (*Sf*N8DT-1), CloQ from *Streptomyces roseochromogenes*, AnaPT from *Neosartorya fischeri* (coAnaPT), NphB from *Streptomyces* sp., and PT from *Streptomyces* sp. Act143 (*Sp*PT), PT from *E. coli* (*Ec*PT), and UbiA from *E. coli*. AnaPT and CdpC3PT from *N. fischeri* without codon optimization were also tested. These PTs are reported in the literature to be able to catalyze the prenylation of several flavonoids.^32–41^ We previously cloned these PTs into the pCDFDuet-1 vector and tested them for the production of prenylnaringenin compounds in *E. coli* M-PAR-121.^42^ The successful expression of these enzymes in *E. coli* M-PAR-121 was previously validated using sodium dodecyl sulfate (SDS) polyacrylamide gel electrophoresis (SDS-PAGE). Only the expression of microbial PTs was observed in the SDS-PAGE gels.^42^

The second step to achieve the final synthesis of xanthohumol was reported to be catalyzed by OMT1 from *H. lupulus* (*Hl*OMT1)^24^ *Hl*OMT1 was previously reported to be responsible for the conversion of desmethylxanthohumol into xanthohumol in hops.^24^ Consequently, *Hl*OMT1 was synthesized with codon optimization and cloned into the pCDFDuet-1 vector. The efficient expression of *Hl*OMT1 in *E. coli* was also assessed using SDS-PAGE, revealing a protein band of the expected size, indicating successful expression of *Hl*OMT1 (Figure S1). Afterward, *Hl*OMT1 was cloned into the previously constructed pCDFDuet-1 vectors holding each PT to further test the production of xanthohumol.^42^ Since naringenin chalcone is expensive, the initial screening of several combinations of PTs with *Hl*OMT1 was performed directly from glucose. The biosynthetic pathway for naringenin chalcone synthesis is well characterized, and the combination of genes was previously optimized and validated in *E. coli* M-PAR-121.^17^ This biosynthetic pathway comprises tyrosine-ammonia lyase (TAL) from *Flavobacterium johnsoniae* (*Fj*TAL), 4-coumarate-CoA ligase 1 (4CL) from *Arabidopsis thaliana* (*At*4CL), and chalcone synthase (CHS) from *Cucurbita maxima* (*Cm*CHS). To reduce the metabolic burden imposed by the expression of several plasmids, a single plasmid holding all of the genes of the naringenin chalcone pathway was constructed (pRSFDuet_*Fj*TAL_*Cm*CHS_*At*4CL). *E. coli* M-PAR-121 expressing pRSFDuet_*Fj*TAL_*Cm*CHS_*At*4CL was tested to validate the production of naringenin chalcone (Figure S2).^17^ This strain was able to produce 479.86 mg/L naringenin chalcone at 63 h. This production was improved to 669.60 mg/L naringenin chalcone after 120 h. This strain was further individually transformed with the 11 pCDFDuet_PT_*Hl*OMT1 vectors. The 11 constructed strains were further tested in 96-deep well plates, and xanthohumol was not detected in any of these strains. The calibration plots for the analyzed metabolites are presented in Figure S3.

*E. coli* M-PAR-121 is a tyrosine-overproducing strain that was previously constructed by Koma et al. to improve the endogenous l-tyrosine production from glucose by integrating eight genes of the shikimate pathway and two genes of central metabolism.^43^ In addition to l-tyrosine, xanthohumol synthesis also depends on DMAPP and SAM availability, as these are needed in the steps catalyzed by PT and OMT enzymes, respectively. The low intracellular availability of both compounds can be the main reason for the absence of desmethylxanthohumol and xanthohumol production in any of the tested pathway combinations (comprising the naringenin chalcone pathway, the different PTs, and *Hl*OMT1) using the wild-type *E. coli* M-PAR-121 strain.^44–46^ The same behavior was also observed in our study of prenylnaringenin compounds production since no production of prenylnaringenin was detected before the optimization of the DMAPP supply in the *E. coli* M-PAR-121 wild-type strain.^42^

### 2.2 Engineering of SAM/DMAPP Pathways in *E. coli* M-PAR-121

Xanthohumol synthesis requires the availability of two extender substrates: DMAPP and SAM. DMAPP is required by PT to perform the extension of naringenin chalcone to produce desmethylxanthohumol. SAM is then used to perform the methylation of desmethylxanthohumol to xanthohumol. Both compounds are naturally synthesized in *E. coli* and are involved in key metabolic processes.^45,47,48^ Considering this, their availability may be limited for use in heterologous production reactions. We previously engineered the DMAPP pathway in *E. coli* M-PAR-121 strains to achieve prenylnaringenin production.^42^ These strains were constructed using clustered regularly interspaced short palindromic repeats (CRISPR)-associated protein 12a (Cas12a) (CRISPR-Cas12a) to perform the integration of heterologous and native 1-deoxy-d-xylulose-5-phosphate synthase (DXS) and isopentenyl diphosphate isomerase (IDI) genes. The heterologous genes DXS from *Bacillus subtilis*, IDI from *S. cerevisiae*, and IDI from *Bacillus licheniformis* were selected. The following eight strains were previously constructed toward the improvement of DMAPP availability and were available to use in this study:^42^ *E. coli* M-PAR-121:*Bs*DXS, *E. coli* M-PAR-121:*Sc*IDI, *E. coli* M-PAR-121:*Bl*DI, *E. coli* M-PAR-121:*Bs*DXS-*Sc*IDI, *E. coli* M-PAR-121:*Bs*DXS-*Bl*IDI, *E. coli* M-PAR-121:*Ec*DXS, *E. coli* M-PAR-121:*Ec*IDI, and *E. coli* M-PAR-121:*Ec*DXS-*Ec*IDI.

Since intracellular SAM levels are also limiting for the OMT1 reaction, improving its availability should also be considered. SAM is natively synthesized in *E. coli* through the action of *metK*.^26^ The overexpression of *metK* in *E. coli* was previously tested by Yu and Zhu, and SAM production was improved.^26^ Thus, the integration of native *E. coli* *metK* into the *E. coli* M-PAR-121 (wild-type strain) and into the eight *E. coli* M-PAR-121 strains with engineered DMAPP pathway was performed using CRISPR-Cas12a.^42^ The following nine strains were successfully constructed using the CRISPR-Cas12a integration system: *E. coli* M-PAR-121:*metK*, *E. coli* M-PAR-121:*Bs*DXS:*metK*, *E. coli* M-PAR-121:*Sc*IDI:*metK*, *E. coli* M-PAR-121:*Bl*DI:*metK*, *E. coli* M-PAR-121:*Bs*DXS-*Sc*IDI:*metK*, *E. coli* M-PAR-121:*Bs*DXS-*Bl*DI:*metK*, *E. coli* M-PAR-121:*Ec*DXS:*metK*, *E. coli* M-PAR-121:*Ec*IDI:*metK*, and *E. coli* M-PAR-121:*Ec*DXS-*Ec*IDI:*metK* (Figure S4).

### 2.3 Evaluation of *De Novo* Xanthohumol Production in the *E. coli* M-PAR-121 Strains with Engineered SAM/DMAPP Pathways

#### 2.3.1 96-Deep Well Plates Screening

The modified *E. coli* strains were transformed with the pRSFDuet_*Fj*TAL_*Cm*CHS_*At*4CL and the pCDFDuet_PT_*Hl*OMT1 vectors. Ninety-nine (99) combinations of strains/pathways were constructed, and their ability to produce desmethylxanthohumol and xanthohumol using glucose as substrate was evaluated in 96-deep well plates (Table 1). Peaks in the same retention time of analytical standards for both desmethylxanthohumol and xanthohumol, or only xanthohumol, were detected in 28 combinations of strains/PTs. As can be observed in Table 1, low productions of both compounds were detected in these production experiments. Desmethylxanthohumol was not detected in some of the combinations, indicating that this compound was fully converted into xanthohumol.

**Table 1:** *De Novo* Production of Desmethylxanthohumol and Xanthohumol in 96-Deep Well Plates Experiments^a^

		Produced compound (μM)			
Strain	PT	CA	NC	DMX	XAN
*E. coli* M-PAR-121:*metK*	*Sf*PT	8.7 ± 0.5	1.8 ± 0.02	-	0.05 ± 0.01
	CdpC3PT	8.3 ± 0.4	2.1 ± 0.1	-	0.05 ± 0.02
	NphB	6.2 ± 0.8	2.5 ± 0.3	0.03 ± 0.005	0.05 ± 0.01
	UbiA	4.4 ± 0.8	2.0 ± 0.2	0.03 ± 0.003	0.03 ± 0.005
*E. coli* M-PAR-121:*Bs*DXS:*metK*	*Sf*PT	4.9 ± 0.6	1.5 ± 0.1	-	0.03 ± 0.001
	AnaPT	71.6 ± 10.5	2.2 ± 0.3	3.5 ± 0.060	0.04 ± 0.003
	CdpC3PT	5.3 ± 0.6	1.5 ± 0.03	-	0.02 ± 0.001
	NphB	5.1 ± 0.8	2.2 ± 0.06	-	0.03 ± 0.002
	*Sp*PT	4.6 ± 0.1	2.6 ± 0.1	0.04 ± 0.019	0.04 ± 0.015
*E. coli* M-PAR-121:*Sc*IDI:*metK*	*Hl*PT1	5.8 ± 0.2	1.8 ± 0.02	-	0.10 ± 0.001
	*Sf*PT	4.7 ± 0.3	1.5 ± 0.02	0.11 ± 0.011	0.05 ± 0.001
	AnaPT	81.9 ± 11.1	2.1 ± 0.2	0.06 ± 0.010	0.03 ± 0.004
	*Ec*PT	4.0 ± 0.6	2.5 ± 0.7	0.05 ± 0.002	0.04 ± 0.002
	NphB	3.4 ± 1.1	1.9 ± 0.1	1.18 ± 0.020	0.04 ± 0.001
	*Sp*PT	4.1 ± 0.6	2.3 ± 0.1	-	0.03 ± 0.002
	UbiA	8.9 ± 0.1	2.4 ± 0.1	0.04 ± 0.015	0.05 ± 0.004
*E. coli* M-PAR-121:*Bl*IDI:*metK*	CdpC3PT	8.3 ± 1.1	1.6 ± 0.03	4.5 ± 0.29	1.5 ± 0.14
*E. coli* M-PAR-121:*Bs*DXS: *Sc*IDI:*metK*	*Ec*PT	6.0 ± 0.6	2.1 ± 0.2	0.02 ± 0.002	0.04 ± 0.001
*E. coli* M-PAR-121:*Bs*DXS: *Bl*IDI:*metK*	*Sf*PT	5.1 ± 1.2	1.5 ± 0.1	-	0.03 ± 0.001
	UbiA	2.5 ± 0.6	1.71 ± 0.09	0.03 ± 0.006	0.03 ± 0.002
*E. coli* M-PAR-121:*Ec*DXS:*metK*	*Hl*PT1	5.6 ± 0.3	1.7 ± 0.02	-	0.09 ± 0.003
	NphB	4.5 ± 1.1	2.0 ± 0.1	-	0.03 ± 0.003
	*Sp*PT	7.1 ± 1.3	2.0 ± 0.03	0.04 ± 0.001	0.03 ± 0.001
*E. coli* M-PAR-121:*Ec*IDI:*metK*	*Sf*PT	4.9 ± 0.2	1.5 ± 0.01	-	0.03 ± 0.006
	*Ec*PT	4.8 ± 0.9	2.0 ± 0.1	-	0.04 ± 0.001
	*Sp*PT	4.9 ± 0.5	2.0 ± 0.1	-	0.03 ± 0.001
	UbiA	7.0 ± 2.3	2.1 ± 0.2	0.03 ± 0.002	0.04 ± 0.003
*E. coli* M-PAR-121:*Ec*DXS:*Ec*IDI:*metK*	UbiA	4.3 ± 0.8	2.4 ± 0.5	0.04 ± 0.006	0.03 ± 0.002

a Only the combinations able to *de novo* produce both compounds or only xanthohumol are herein represented. The experiments were performed in triplicate for each strain/pathway combination. Ultra-high-performance liquid chromatography (UHPLC) was performed to evaluate the production of *p*-coumaric acid (CA), naringenin chalcone (NC), desmethylxanthohumol (DMX), and xanthohumol (XAN).

The best producing strain in these experiments was *E. coli* M-PAR-121:*Bl*IDI:*metK* expressing pRSFDuet_*Fj*TAL_*Cm*CHS_*At*4CL and pCDFDuet_CdpC3PT_*Hl*OMT1. This strain was able to produce 4.5 ± 0.29 μM of desmethylxanthohumol and 1.5 ± 0.14 μM of xanthohumol. In these 96-deep well experiments, *p-*coumaric acid and naringenin chalcone were produced and accumulated only in residual amounts, which limits further production of desmethylxanthohumol and xanthohumol. Although these experiments using 96-deep well plates are useful for a fast screening of several conditions in parallel, these plates are not the optimal platform to promote cellular growth since the airspace and, consequently, oxygen transfer are limited. The airspace ratio used in 96-deep well plates is 1:2 since the 2 mL plate wells were filled with 1 mL of culture. In contrast, a 1:5 airspace ratio is used in shake flask experiments (50 mL of culture in a 250 mL shake flask), promoting cellular growth. In addition to the airspace limitation, the integration and overexpression of two or more genes, as well as the expression of a biosynthetic pathway composed of five genes, are probably impairing the cellular growth and, consequently, the production of the heterologous compounds due to a high metabolic burden of the cells. Additionally, SAM synthesis, catalyzed by the *metK* gene product, is dependent on adenosine triphosphate (ATP).^49^ ATP is essential for several *E. coli* metabolic pathways and an imbalance in the ATP utilization can affect the cell’s homeostasis and growth.^50^ In fact, it was verified in these experiments that *E. coli* strains with engineered DMAPP/SAM pathways took longer to grow and reach the target optical density at 600 nm (OD_600nm_) for the induction of protein expression, compared with the *E. coli* strains with the engineered DMAPP pathway (*data not shown*).

#### 2.3.2 Shake Flasks Experiments

To validate production levels achieved in 96-deep well experiments, all 28 identified producing strains were tested in shake flask experiments. Production experiments were carried out using a combination of LB Miller and M9 minimal medium previously optimized to produce the intermediate naringenin chalcone.^17^ LB Miller medium is used for cell growth and initial protein expression. Then, the culture is changed to an M9 minimal medium that contains the substrate for the production experiment. The production of all of the metabolites by the 28 identified producing strains was evaluated (Table 2). In these experiments, a higher production of *p-*coumaric acid was obtained compared with production performed in 96-deep well plates. This suggests that using shake flasks is beneficial for growth due to greater airspace and better oxygen transfer and that this enhances the production of the first intermediate of the pathway, *p-*coumaric acid. However, *p-*coumaric acid was accumulated, and it was not converted to naringenin chalcone since the production levels were similar to those achieved in 96-well experiments.

**Table 2:** *De Novo* Production of Desmethylxanthohumol and Xanthohumol in Shake Flask Experiments Using the Combination of LB Miller and M9 Minimal Media^a^

		Produced compound (μM)			
Strain	PT	CA	NC	DMX	XAN
*E. coli* M-PAR-121:*metK*	*Sf*PT	677.6 ± 33.1	1.8 ± 0.1	0.20 ± 0.02	0.07 ± 0.013
	CdpC3PT	656.7 ± 58.6	3.7 ± 0.1	-	0.03 ± 0.003
	NphB	635.0 ± 30.3	1.6 ± 0.04	-	0.10 ± 0.01
	UbiA	254.8 ± 15.1	1.4 ± 0.03	0.04 ± 0.002	0.04 ± 0.004
*E. coli* M-PAR-121:*Bs*DXS:*metK*	*Sf*PT	723.1 ± 72.8	2.0 ± 0.3	0.05 ± 0.02	0.10 ± 0.02
	AnaPT	308.2 ± 15.8	1.7 ± 0.1	0.08 ± 0.02	-
	CdpC3PT	513.1 ± 75.8	1.8 ± 0.1	-	0.03 ± 0.003
	NphB	484.9 ± 35.5	1.5 ± 0.1	-	-
	*Sp*PT	805.4 ± 36.3	1.6 ± 0.3	-	0.03 ± 0.005
*E. coli* M-PAR-121:*Sc*IDI:*metK*	*Hl*PT1	230.5 ± 40.4	1.6 ± 0.01	0.04 ± 0.001	0.03 ± 0.002
	*Sf*PT	295.5 ± 16.9	1.6 ± 0.01	0.04 ± 0.001	0.02 ± 0.004
	AnaPT	513.9 ± 60.5	1.7 ± 0.1	0.03 ± 0.001	0.03 ± 0.004
	*Ec*PT	419.8 ± 31.9	1.5 ± 0.01	-	0.03 ± 0.01
	NphB	486.0 ± 35.5	1.7 ± 0.1	-	0.06 ± 0.01
	*Sp*PT	386.8 ± 82.0	1.4 ± 0.1	-	0.03 ± 0.005
	UbiA	295.7 ± 38.8	1.4 ± 0.1	-	0.11 ± 0.004
*E. coli* M-PAR-121:*Bl*IDI:*metK*	CdpC3PT	1613.0 ± 203.5	2.6 ± 0.2	0.06 ± 0.02	1.5 ± 0.2
*E. coli* M-PAR-121:*Bs*DXS:*Sc*IDI:*metK*	*Ec*PT	807.2 ± 33.5	2.1 ± 0.1	0.04 ± 0.002	0.11 ± 0.007
*E. coli* M-PAR-121:*Bs*DXS:*Bl*IDI:*metK*	*Sf*PT	402.1 ± 20.9	2.1 ± 0.5	0.03 ± 0.002	0.13 ± 0.03
	UbiA	62.0 ± 5.0	1.4 ± 0.01	-	0.09 ± 0.001
*E. coli* M-PAR-121:*Ec*DXS:*metK*	*Hl*PT1	106.7 ± 0.6	1.8 ± 0.2	0.04 ± 0.002	0.02 ± 0.002
	NphB	569.2 ± 1.7	1.6 ± 0.3	0.17 ± 0.03	0.188 ± 0.20
	*Sp*PT	601.3 ± 42.4	-	-	-
*E. coli* M-PAR-121:*Ec*IDI:*metK*	*Sf*PT	260.7 ± 20.8	1.7 ± 0.04	0.04 ± 0.01	0.03 ± 0.02
	*Ec*PT	38.1 ± 2.3	1.6 ± 0.2	0.13 ± 0.05	-
	*Sp*PT	236.4 ± 42.1	2.1 ± 0.6	-	0.25 ± 0.06
	UbiA	3.6 ± 0.1	1.3 ± 0.02	-	-
*E. coli* M-PAR-121:*Ec*DXS:*Ec*IDI:*metK*	UbiA	22.9 ± 6.9	1.4 ± 0.003	-	-

a The experiments were performed in triplicate for each identified producing strain/pathway combination. Ultra-high-performance liquid chromatography (UHPLC) was performed to evaluate the production of *p-*coumaric acid (CA), naringenin chalcone (NC), desmethylxanthohumol (DMX), and xanthohumol (XAN).

As occurred in 96-deep well plate experiments, the highest production levels were achieved in *E. coli* M-PAR-121:*Bl*IDI:*metk* expressing pRSFDuet_*Fj*TAL_*Cm*CHS_*At*4CL and pCDFDuet_CdpC3PT_*Hl*OMT1. This strain was able to produce 0.06 ± 0.02 μM of desmethylxanthohumol and 1.5 ± 0.12 μM of xanthohumol. Desmethylxanthohumol production was lower than the production achieved in the first screening. However, the production of xanthohumol was in the same range. Since this strain/pathway combination showed the highest xanthohumol production in 96-deep well plates and shake flask experiments using LB+M9, this strain/pathway was chosen for further study to optimize production.

For larger-scale production, it is preferable to use a single production medium and perform the process in one step. Shake flask experiments for the best producing strain were performed using M9-modified medium and TB medium (Figure 2). The TB recipe was the same as the one previously used in 96-deep well plate experiments. On the other hand, M9-modified medium was altered by replacing the vitamins solution with trace elements and yeast extract to promote bacterial growth and improve protein expression.^51–53^

**Figure 2:**
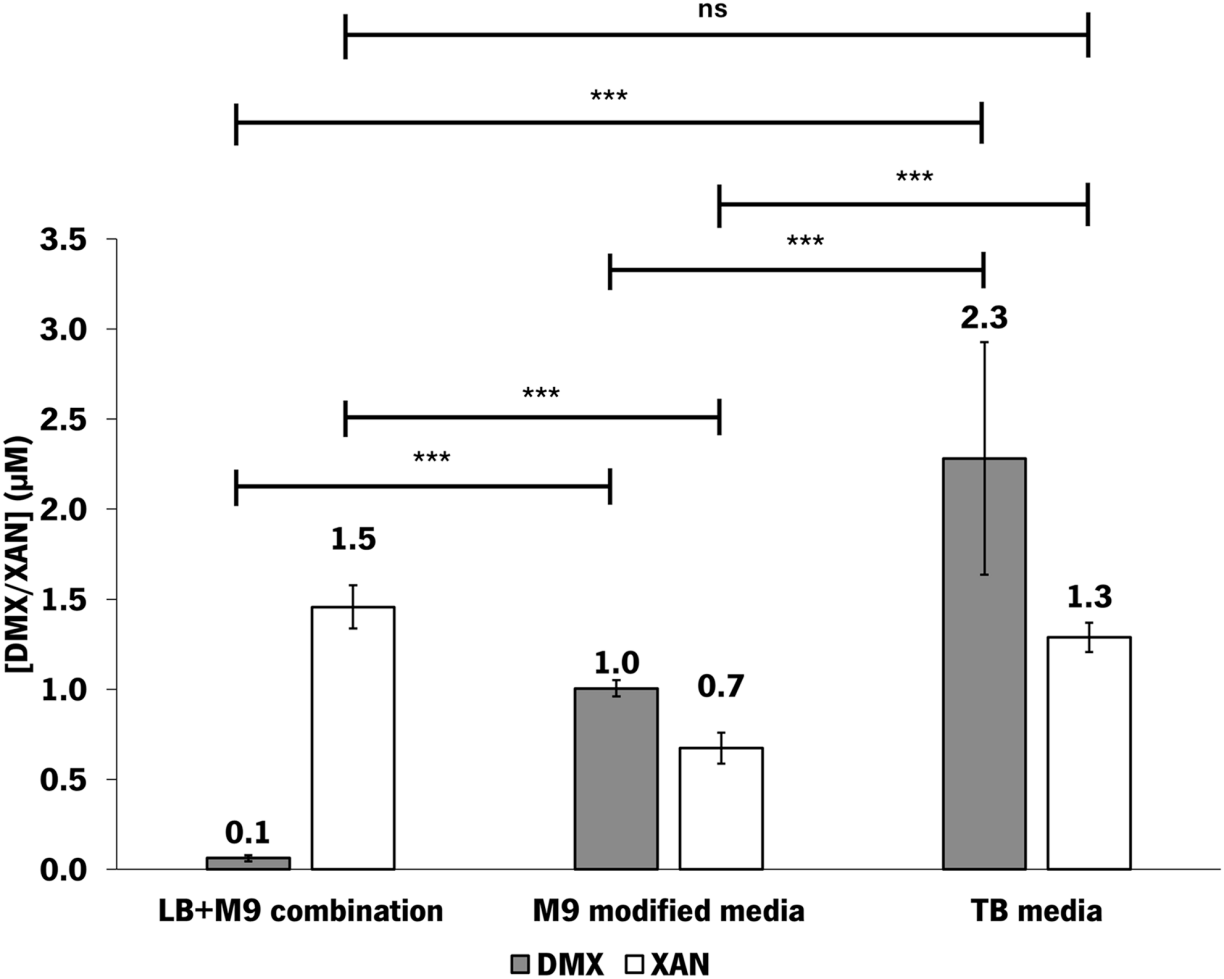
*De novo* production of desmethylxanthohumol (DMX) and xanthohumol (XAN) by *E. coli* M-PAR-121:*Bl*IDI:*metK* expressing pRSFDuet_*Fj*TAL_*Cm*CHS_*At*4CL and pCDFDuet_CdpC3PT_*Hl*OMT1 in shake flask experiments using different production media. The combination of LB+M9 and the use of only M9-modified medium and TB medium were tested. The production experiments were carried out using glucose as the sole substrate. Results correspond to the average of three independent experiments ± the standard deviation.

As can be observed in Figure 2, the production of desmethylxanthohumol was statistically significantly higher (*p-*values <0.05 using Tukey’s multiple comparisons test) than the one achieved in the LB+M9 experiment, both in M9-modified and TB experiments. Regarding the production using TB, desmethylxanthohumol production was significantly higher compared to that obtained in LB+M9 and M9-modified medium (*p-*values <0.05 using Tukey’s multiple comparisons test). Regarding xanthohumol, the production attained was significantly lower using M9-modified medium. This result indicates that M9-modified medium does not favor the methylation reaction catalyzed by *Hl*OMT1 compared with the combined use of LB+M9. Moreover, the production of xanthohumol in TB was in the same range as production in LB+M9 (*p-*value >0.05 using Tukey’s multiple comparisons test). The higher desmethylxanthohumol accumulation in the TB experiment indicates that this compound is being produced in higher amounts and is not being successfully converted to xanthohumol by *Hl*OMT1, possibly due to limiting *Hl*OMT1 activity.

Production of all of the metabolites was evaluated throughout the experiment (Figure 3). As can be observed in Figure 3, *p-*coumaric acid and naringenin chalcone production in LB+M9 reached 161.3 and 25.6 μM, respectively. These production levels were almost constant from 48 h until the end of the experiment. Contrary to the other experiments, xanthohumol was first detected at 48 h. In the experiment using M9-modified medium, 270.6 μM *p-*coumaric acid and 10.5 μM naringenin chalcone were produced. Regarding desmethylxanthohumol and xanthohumol, these compounds were detected after 48 and 72 h, respectively. However, higher amounts of desmethylxanthohumol were accumulated without being converted to xanthohumol. In the experiment using TB, lower amounts of *p-*coumaric acid (18.6 μM) were produced and accumulated. For scaled production, lower accumulation of the intermediate *p-*coumaric acid can be useful since production of a less complex mixture of compounds lessens downstream processing/purification costs. However, higher amounts of naringenin chalcone were detected (44.7 μM). Desmethylxanthohumol and xanthohumol were detected after 48 and 72 h, respectively. As occurred in M9-modified medium, higher amounts of desmethylxanthohumol were accumulated without being converted to xanthohumol. That said, the production of xanthohumol obtained in this medium was in the range of the production achieved in LB+M9. Moreover, higher amounts of xanthohumol were detected sooner in the experiment being important for the productivity of the process.

**Figure 3:**
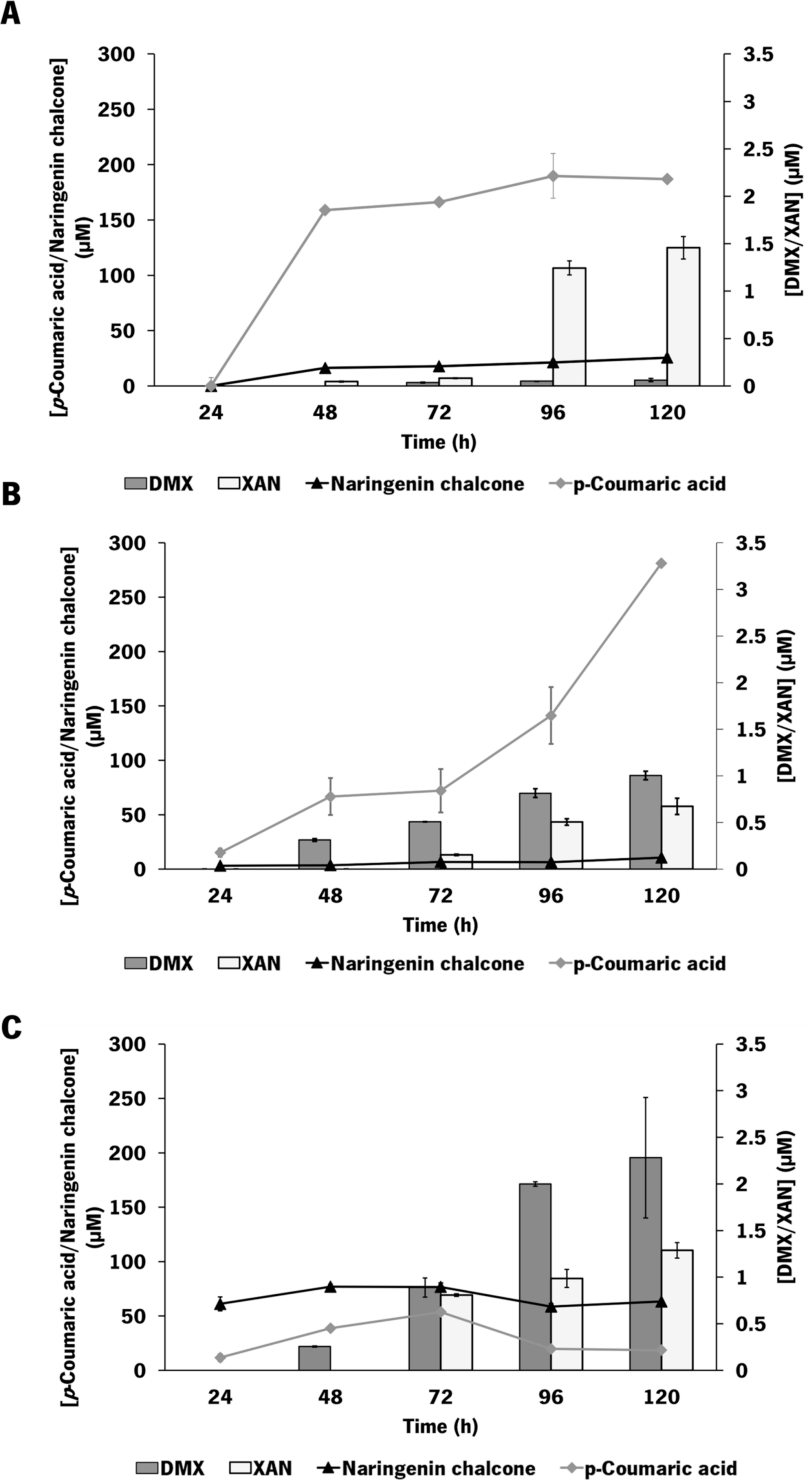
Profile of *p*-coumaric acid, naringenin chalcone, desmethylxanthohumol (DMX), and xanthohumol (XAN) production by *E. coli* M-PAR-121:*Bl*IDI:*metK* expressing pRSFDuet_*Fj*TAL_*Cm*CHS_*At4*CL and pCDFDuet_CdpC3PT_*Hl*OMT1. Shake flask experiments were performed by using LB+M9 (A), M9-modified medium (B), and TB medium (C). Results correspond to the average of three independent experiments ± standard deviation.

Considering the production levels of desmethylxanthohumol and xanthohumol attained in TB, this medium was selected for 2 L lab-scale stirring bioreactor experiments.

#### 2.3.3 Bioreactor Scale Experiments

*E. coli* M-PAR-121:*Bl*IDI:*metK* expressing pRSFDuet_*Fj*TAL_*Cm*CHS_*At*4CL and pCDFDuet_CdpC3PT_*Hl*OMT1 was also tested at a 2 L lab-scale stirring bioreactor scale using TB medium. In a first approach, a batch experiment was conducted using 30 g/L glucose as the substrate (Figure 4).

**Figure 4:**
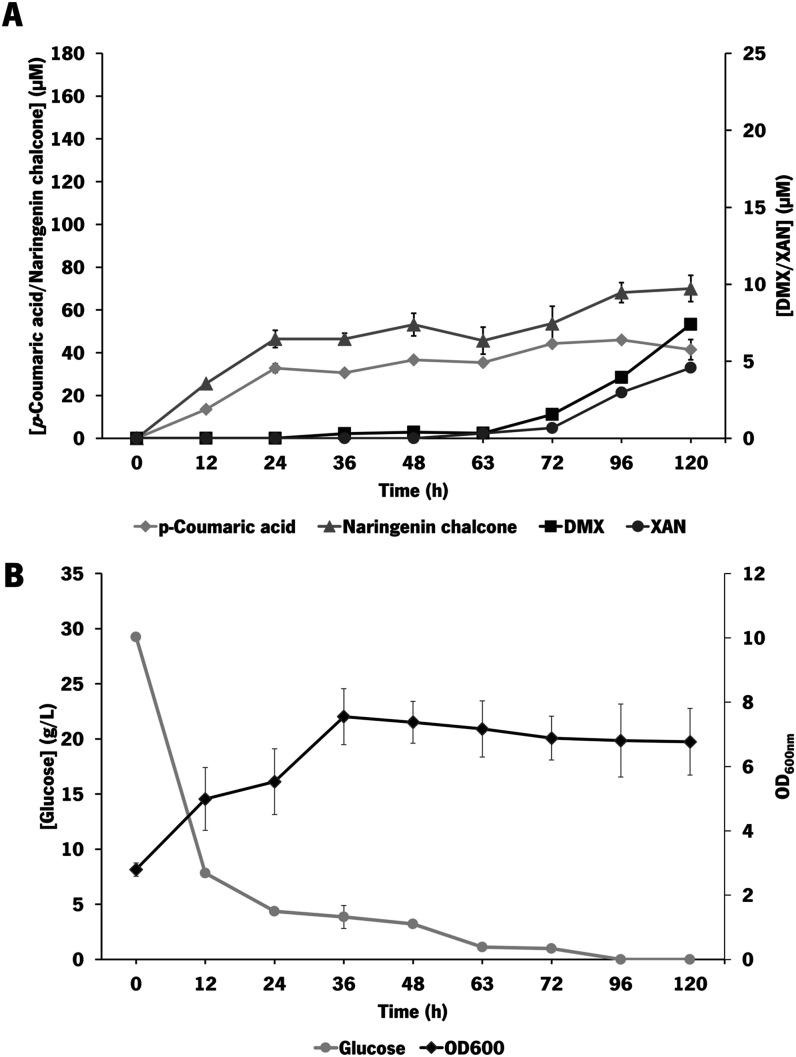
Evaluation of metabolites accumulation, glucose consumption, and cell growth in the bioreactor batch experiment by *E. coli* M-PAR-121:*Bl*IDI:*metK* expressing pRSFDuet_*Fj*TAL_*Cm*CHS_*At*4CL and pCDFDuet_CdpC3PT_*Hl*OMT1. (A) Profile of *p-*coumaric acid, naringenin chalcone, desmethylxanthohumol (DMX), and xanthohumol (XAN) production and accumulation. (B) Glucose consumption and optical density at 600 nm (OD_600nm_) of the strain during the experiment. Results correspond to the average of two independent experiments ± the standard deviation.

Regarding the production of desmethylxanthohumol and xanthohumol, the production of both compounds was increased throughout the experiment, with higher levels between 96 and 120 h. Desmethylxanthohumol and xanthohumol were detected for the first time at 36 and 63 h, respectively. At the final time point, 7.4 μM (2.5 mg/L) desmethylxanthohumol and 4.6 μM (1.6 mg/L) xanthohumol were produced. Compared to the shake flask experiments, the improvement in production levels of desmethylxanthohumol and xanthohumol was statistically significant, using the unpaired *t*-test with Welch’s correction test, corresponding to a 3.25-fold (*p-*value of 0.0376) and 3.55-fold improvement (*p-*value of 0.0084), respectively. Moreover, almost 27 g/L of glucose was consumed in the first 36 h of the experiment, corresponding also to the period of higher growth of the strain. After that, the strain growth was maintained almost constant, probably due to the absence of a carbon source, reaching a final OD_600nm_ of 6.9 (Figure 4B). The higher cell growth attained in the bioreactor compared with the shake experiments (OD_600nm_ of 6.9 vs OD_600nm_ of 2.9) is probably the main reason for the improvement in the production of all of the metabolites

Considering the profile of glucose consumption, another experiment was carried out by supplementing with two additional pulses of glucose (10 g/L) at 24 and 48 h. Due to the higher glucose concentration, this experiment was kept for 144 h (Figure 5).

**Figure 5:**
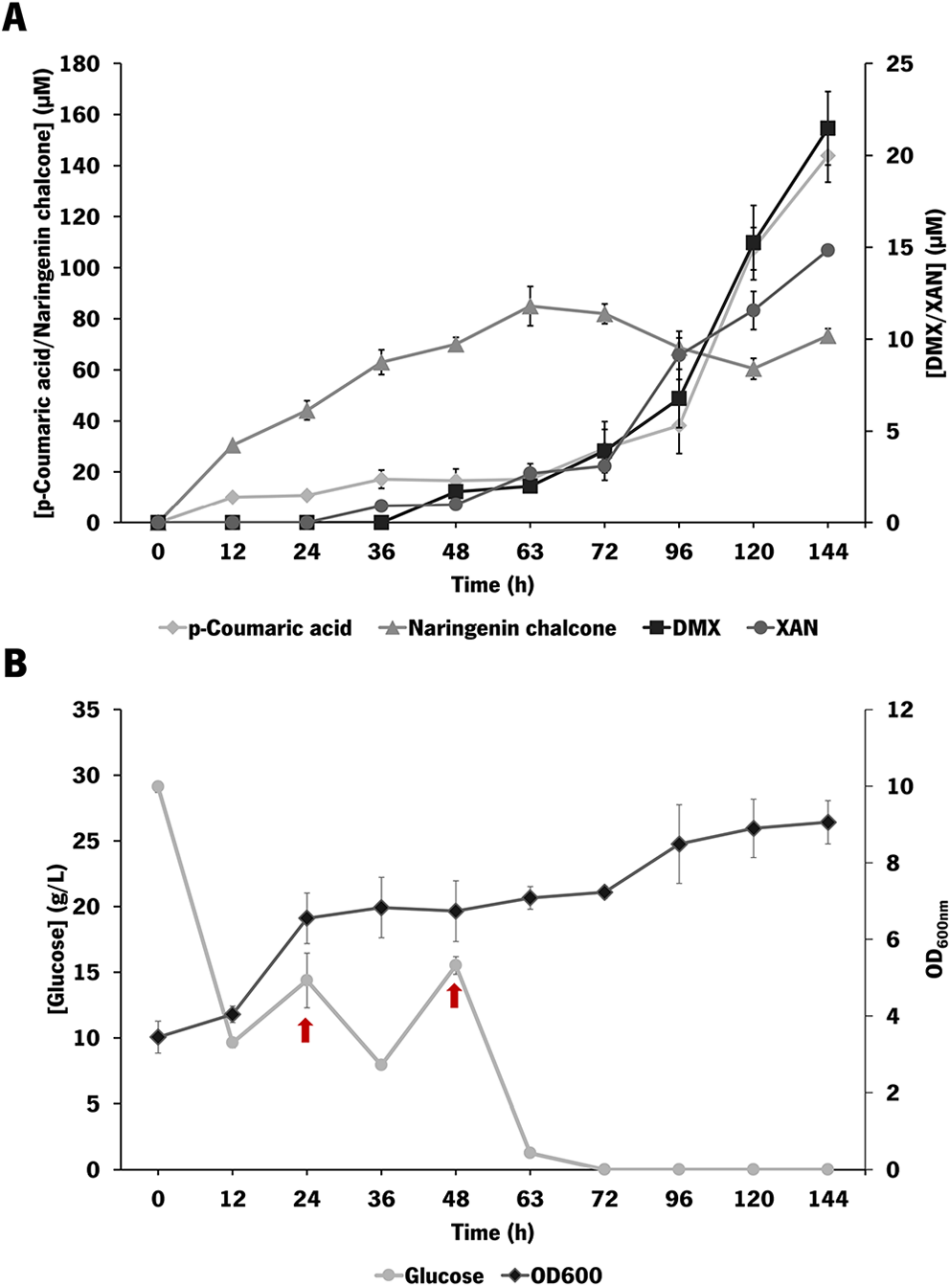
Evaluation of metabolites accumulation, glucose consumption, and cell growth in the batch experiment with two additional glucose pulses (10 g/L) in a bioreactor by *E. coli* M-PAR-121:*Bl*IDI:*metK* expressing pRSFDuet_*Fj*TAL_*Cm*CHS_*At*4CL and pCDFDuet_CdpC3PT_*Hl*OMT1. (A) Profile of *p-*coumaric acid, naringenin chalcone, desmethylxanthohumol (DMX), and xanthohumol (XAN) production and accumulation. (B) Glucose consumption and optical density at 600 nm (OD_600nm_) of the strain during the experiment. Two additional pulses of 10 g/L glucose, represented by a red arrow, were added at 24 and 48 h. Results correspond to the average of two independent experiments ± standard deviation.

As can be observed in Figure 5A, the tendency for a higher accumulation of naringenin chalcone than *p-*coumaric acid was also maintained in this experiment during almost the entire time frame. However, *p-*coumaric acid production significantly increased in the two final time points of the experiment, reaching 143.9 μM. Also, 73.3 μM of naringenin chalcone was detected at the final time point. The production levels of both compounds were higher than those achieved in the first bioreactor experiment (Figure 4A). Regarding the production, the levels of desmethylxanthohumol and xanthohumol continuously increased throughout the experiment. Contrary to the first bioreactor experiment, xanthohumol was first detected at 36 h, and desmethylxanthohumol was only detected at 48 h, indicating that the produced desmethylxanthohumol was immediately converted into xanthohumol in the first 36 h. Higher amounts of both compounds were accumulated after 63 h of the experiment. At the final time point, 21.5 μM desmethylxanthohumol (7.3 mg/L) and 14.8 μM xanthohumol (5.3 mg/L) were accumulated. Despite the addition of two additional glucose pulses, this carbon source was completely consumed within 72 h (Figure 5B). However, the culture growth was improved, reaching a final OD_600nm_ of 9.1, probably being the main reason for the higher production and accumulation of metabolites. A representative UHPLC chromatogram of this sample and the reference standard is presented in Figure S5. These titers represent a 1.5-fold and 2.8-fold increase compared to productions achieved without the additional glucose pulses, and these are the highest production levels reported so far. To the best of our knowledge, only *S. cerevisiae* has been engineered to produce these compounds by expressing *Hl*PT1 and *Hl*OMT1 in combination with the naringenin chalcone biosynthetic pathway composed of *Fj*TAL and 4CL, CHS, and noncatalytic CHI from *H. lupulus*. Following optimization to improve l-tyrosine availability, DMAPP flux, and PT catalytic activity, 2.2 mg/L of desmethylxanthohumol and 0.14 mg/L of xanthohumol from glucose were produced in shake flask experiments using 20 g/L glucose as substrate. However, no modifications were performed toward the improvements of SAM availability and OMT1 activity.^28^

Like most aromatic PTs, CdpC3PT from *N. fischeri* has a wide catalytic versatility, being able to perform the prenylation reaction in different positions depending on the substrate, mostly recognized to perform C3 prenylation in the indole ring of tryptophan derivatives.^54–59^ Due to the absence of *in vitro* investigation of these enzymes, including CdpC3PT, using naringenin chalcone as substrate due to its high cost and limited availability, further investigation should be performed to confirm the structure of the produced metabolites. In a first analysis, the UV spectra of the desmethylxanthohumol and xanthohumol peaks in this sample were compared with the UV spectra of the peaks in the reference standard (Figure S6). As can be observed in the figure, both desmethylxanthohumol and xanthohumol peaks present similar UV spectra profiles compared with the respective peaks in the reference standard. Given the demonstrated efficiency and accuracy of our UHPLC method in separating isomers with prenyl groups at different positions,^42^ we consider it highly likely that the product obtained corresponds to the expected compound and not to another potential isomer. This conclusion is supported by the consistency observed in both retention time and UV spectra when compared with authentic standards of desmethylxanthohumol and xanthohumol. Nevertheless, the reference standards and the final sample from the bioreactor experiment were analyzed by liquid chromatography–mass spectrometry (LC–MS) to allow the structural identification of the produced compounds. The standards were first analyzed in the selected ion monitoring (SIM) mode, with the results summarized in Table S7. They were subsequently analyzed in MS/MS (MS^2^) mode to obtain the fragmentation spectra of each compound (Figure S7). Subsequently, the sample was analyzed in MS^2^ mode, and the obtained spectra were compared with those of the reference standards (Figure S8). The characteristic fragments of each compound found in the sample in the MS^2^ spectrum (30% normalized collision energy (NCE)) are also presented in Table S8. A clear match between the sample peaks and the reference standards in both precursor ions and diagnostic fragment ions was found. These results confirm that the main end products are indeed desmethylxanthohumol and xanthohumol.

In our study, high levels of naringenin chalcone were produced and were not converted into desmethylxanthohumol in all experiments. This suggests that the prenylation step is a limiting factor for the efficient production of desmethylxanthohumol. The same behavior was also observed in the production of several prenylated compounds both in *E. coli* and in *S. cerevisiae*, including prenylnaringenin compounds and desmethylxanthohumol.^28,42,60–65^ Consequently, it will be important to further improve PT activity and find alternative strategies to improve DMAPP availability. Regarding PT activity, strategies of rational design and random or directed mutagenesis of key amino acids involved in the catalysis and in the interactions with donor and acceptor should be considered to enhance the enzyme catalytic activity.^28,66^ Regarding DMAPP improvement, the reconstruction of the heterologous mevalonate (MVA) pathway can be tested along with other strategies to downregulate competing pathways that divert DMAPP^67,68^

Moreover, it was also verified that higher amounts of desmethylxanthohumol in comparison to xanthohumol were accumulated. This behavior was also observed by Yang et al. for xanthohumol production in *S. cerevisiae*. Overall, this higher desmethylxanthohumol accumulation suggests that the methylation step, catalyzed by *Hl*OMT1, is one of the rate-limiting steps in this biosynthetic pathway and that should be improved to achieve higher production of xanthohumol. This can occur due to a limited activity of *Hl*OMT1 and/or due to low availability of the intracellular SAM despite *metK* being integrated and overexpressed in the *E. coli* genome. Considering this, alternative OMT enzymes from plants and from microbial sources can be tested.^69–71^ Moreover, as suggested for PTs, strategies to enhance the catalytic activity of the enzyme should be exploited.^69^ Alternative strategies can also be exploited to improve SAM availability, namely the construction of the betaine utilization pathway that has already been exploited in *E. coli*.^72^

All of these modifications should be considered to improve the production levels herein reported, since they are still far from the required amounts to meet the industrial demand. However, as far as we know, our work represents the first report of *de novo* production of xanthohumol using *E. coli* as a microbial cell factory and the highest production level reported so far in any microbial chassis.

## 3 Methods

### 3.1 Strains, Plasmids, Chemicals, and Media Composition

Cloning and the plasmids maintenance and propagation were performed in *E. coli* NZY5α (NZYTech-MB00401) and *E. coli* NEB5α (New England Biolabs-C2987H). *E. coli* M-PAR-121 (wild-type strain) was kindly provided by Koma et al.^43^ *E. coli* M-PAR-121 with modifications to improve the availability of DMAPP was used to perform CRISPR-Cas12a modifications toward the improvement of SAM availability.^42^ These strains were used for the expression of heterologous biosynthetic pathways to produce xanthohumol. The features of all of these strains are presented in Table S1.

All of the plasmids used and constructed in this study are presented in Table S2. AnaPT and CdpC3PT from *N. fischeri* were previously amplified from pWY16 and pWY24, which were kindly provided by Dr. Li.^57,73^ The plasmids used in CRISPR-Cas12a strategies (pSIM*cpf1* and pTF-*ahpC-rfp*) were designed and kindly provided by Jervis et al.^74^

LB agar (20 g/L LB Lennox (LabKem) and 20 g/L agar (LabKem)) was used for colonies, strain selection, and maintenance. Production experiments were performed using lysogeny broth (LB) Miller medium (NZYTech) combined with M9 minimal medium, M9-modified medium, and TB medium. Glucose (Acros) was supplemented to the media to be used as a substrate at a final concentration of 30 g/L. Isopropyl β-d-1-thiogalactopyranoside (IPTG) (NZYTech) was supplemented to induce protein expression at a final concentration of 0.1 mM. l-arabinose (0.2% (w/v)) (Enzymatic) was used for the curing of CRISPR plasmids. Strain selection was performed using antibiotics: spectinomycin (Alfa Aesar) (100 or 50 μg/mL), kanamycin (NZYTech) (50 μg/mL), chloramphenicol (NZYTech) (25 μg/mL), and hygromycin (Fischer Scientific) (150 μg/mL).

M9 minimal medium composition was 6 g/L Na_2_HPO_4_ (Chem-Lab), 5 g/L CaCO_3_ (Panreac), 3 g/L KH_2_PO_4_ (Riel-deHaën), 1 g/L NH_4_Cl (Panreac), 0.5 g/L NaCl (NZYTech), 340 mg/L thiamine (Thermo Fisher Scientific), 110 mg/L MgSO_4_ (Labkem), 15 mg/L CaCl_2_ (Panreac), and vitamins. The following vitamins were included: 12.2 mg/L nicotinic acid (Acros Organics), 2.8 mg/L pyridoxine (Fisher BioReagents), 10.8 mg/L pantothenic acid (Sigma-Aldrich), 0.12 mg/L biotin (Merck), 0.84 mg/L riboflavin (Panreac), and 0.084 mg/L folic acid (Panreac).

Terrific broth (TB) was composed of 24 g/L yeast extract, 12 g/L tryptone (Fisher Scientific), 9.4 g/L KH_2_PO_4_, and 2.2 g/L K_2_HPO_4_ (Panreac).

M9-modified medium was composed of 6 g/L Na_2_HPO_4_, 5 g/L yeast extract (LabKem), 3 g/L KH_2_PO_4,_ 1 g/L NH_4_Cl, 0.5 g/L NaCl, 110 mg/L MgSO_4_,15 mg/L CaCl_2_, 270 mg/L FeCl_3_·6H_2_O (Panreac) and trace elements. Trace elements included 200 mg/L CoCl_2_·6H_2_O (Sigma-Aldrich), 200 mg/L ZnCl_2_·4H_2_O (LabKem), 200 mg/L Na_2_MoO_4_·2H_2_O (Acros), 13 mg/L CuCl_2_·6H_2_O (Sigma-Aldrich), 5 mg/L H_3_BO_3_ (Fisher Scientific), and 0.1 mL/L HCl (Fisher Scientific).

### 3.2 Construction of the Pathway Plasmids

First, a single plasmid carrying the previously optimized naringenin chalcone biosynthetic pathway composed of *Fj*TAL, *At*4CL, and *Cm*CHS was constructed.^17^ The previously constructed pRSFDuet_*Fj*TAL_*Cm*CHS vector^17^ was linearized by polymerase chain reaction (PCR). *At*4CL and the respective promoter and terminator were amplified by PCR using as template pACYCDuet_*At*4CL^75^ and primers with 15 bp homology overhangs for the pRSFDuet_*Fj*TAL_*Cm*CHS. The fragments were assembled using the In-Fusion Snap Assembly kit (Takara Bio Europe). The correct construction of pRSFDuet_*Fj*TAL_*Cm*CHS_*At*4CL was confirmed by colony PCR and sequencing (Eurofins).

Eleven PTs previously selected for prenylnaringenin production were also tested in this study to catalyze the conversion of naringenin chalcone into desmethylxanthohumol.^42^ *Hl*OMT1 was selected to catalyze the last step of the xanthohumol biosynthetic pathway responsible for the conversion of demethylxanthohumol into xanthohumol.^24^ This gene was codon-optimized for *E. coli* by Twist Bioscience (Table S3) and amplified by PCR using primers with 15 bp homology overhangs for the pCDFDuet vector holding each PT. All of these vectors were linearized by PCR using the same pair of primers for fragment insertion into the multiple cloning site (MCS) 2 of the vector, since all of the PTs are cloned into the MCS 1. Cloning was performed by In-Fusion using the In-Fusion Snap Assembly kit. The constructions were confirmed by colony PCR and sequencing. The used primers (Metabion/Eurofins) are displayed in Table S4.

### 3.3 Evaluation of OMT Expression

*E. coli* M-PAR-121 carrying the pCDFDuet_*Hl*OMT1 was grown at 37 °C and 200 rpm in an LB Miller. When an OD_600nm_ of 0.6 was attained, 0.1 mM IPTG was added to induce protein expression. The culture was incubated at 26 °C and 200 rpm for 6 h. Samples were taken before induction (0 h sample) and 6 h after induction (6 h sample). These samples were centrifuged, and the cell pellets were resuspended in 10 mM Tris-HCl buffer (pH 7.8). Cells were disrupted by using a microtip probe linked to a Vibra-cell processor (Sonics). Samples preparation, protein quantification, and the evaluation by SDS-PAGE were performed as described in Gomes et al.^17^ NZYColour Protein Marker II (NZYTech) was used as a reference protein ladder.

### 3.4 Engineering *E. coli* toward the Improvement of the Extender Substrate SAM

*E. coli* M-PAR-121 and the *E. coli* previously constructed *E. coli* M-PAR-121 strains with engineered DMAPP pathway^42^ were used to integrate an additional copy of *metK* from *E. coli* sorting to the CRISPR-Cas12a system.^74^ The integration of *metK* was performed to improve SAM availability, which is the extender substrate required in the OMT1 step.

Since the DMAPP modifications (heterologous and native DXS and IDI integrations) were previously introduced into the β-galactosidase (*lacZ*) locus of the genome,^42^ the alternative alkyl hydroperoxide reductase C (*ahpC*) locus was selected to perform *metK* integration. The sequence of the native *metK* gene is presented in Table S5. The pTF-*ahpC*-*rfp* vector constructed by Jervis et al. was used. This vector was linearized by PCR to remove the red fluorescence protein coding gene (*rfp)* donor DNA used by Jervis et al., using primers annealing into the extremities of the *ahpC* arms.^74^ Linearized pTF-*ahpC* was used as the backbone to receive the *metK* for genome integration. The *metK* gene was amplified by PCR using *E. coli* M-PAR-121 genomic DNA as a template and primers containing 15 bp homology arms for the linearized pTF-*ahpC* vector. The fragments were assembled using the In-Fusion Snap Assembly kit. The target-specific integration vector pTF-*ahpC-metK* was constructed. The construction was confirmed by colony PCR and sequencing. The primers (Metabion/Eurofins) used are displayed in Table S6.

Chemically competent cells of *E. coli* M-PAR-121 and of *E. coli* M-PAR-121 strains with engineered DMAPP pathway were first transformed with the pSIM*cpf1* vector that contains all of the machinery required for CRISPR-Cas12 integration and plasmid curing.^74^ Afterward, electrocompetent cells of these transformants were prepared, and pTF-*ahpC-metK* was electroporated. Recombinants were selected in LB agar with hygromycin (150 μg/mL) and spectinomycin (50 μg/mL) at 30 °C and then grown overnight for further genomic DNA extraction using Monarch Genomic DNA Purification Kit (NEB). The extracted genomic DNA was used as a template to confirm the integration by PCR. Integration was also confirmed by sequencing. Plasmid curing was performed as described by Jervis et al.^74^ The following strains were constructed: *E. coli* M-PAR-121:*metK*, *E. coli* M-PAR-121:*Bs*DXS:*metK*, *E. coli* M-PAR-121:*Sc*IDI:*metK*, *E. coli* M-PAR-121:*Bl*DI:*metK*, *E. coli* M-PAR-121:*Bs*DXS-*Sc*IDI:*metK*, *E. coli* M-PAR-121:*Bs*DXS-*Bl*IDI:*metK*, *E. coli* M-PAR-121:*Ec*DXS:*metK*, *E. coli* M-PAR-121:*Ec*IDI:*metK*, and *E. coli* M-PAR-121:*Ec*DXS-*Ec*IDI:*metK*.

### 3.5 Production Experiments

*E. coli* M-PAR-121 (wild-type strain) and *E. coli* M-PAR-121 strains with engineered DMAPP/SAM pathways were transformed by the heat-shock method with the plasmid carrying the naringenin chalcone pathway (pRSFDuet_*Fj*TAL_*At*4CL_*Cm*CHS) and the plasmid containing each PT in combination with *Hl*OMT1. The transformants were selected in LB agar with 50 μg/mL kanamycin and 100 μg/mL spectinomycin.

#### 3.5.1 96-Deep Well Plates Experiments

Overnight grown cultures of the transformant strains were freshly cultivated (1:100) in 1 mL of TB containing 4 g/L of glucose and the respective antibiotics in 96-deep well plates. Plates were sealed with a breathable sealing film to ensure oxygen transference. The plate was incubated at 37 °C and 1000 rpm. When an OD_600nm_ between 1.5 and 2.0 was achieved, 0.1 mM IPTG was added. The cultures were then incubated at 30 °C and 1000 rpm. After 2 h, 30 g/L of glucose was supplemented to be used as substrate, and the plate was incubated at 30 °C and 1000 rpm for 120 h. These experiments were performed in triplicate.

#### 3.5.2 Shake Flask Experiments

The producing strains selected in 96-deep well plates experiments were then evaluated in shake flask experiments. Overnight grown cultures were used to inoculate 50 mL of LB Miller supplemented with 50 μg/mL of kanamycin and 100 μg/mL of spectinomycin (initial OD_600nm_ of 0.1). The cultures were grown at 37 °C and 200 rpm, until an OD_600nm_ of 0.9. Then, 0.1 mM IPTG was added to induce the protein expression. After 5 h of incubation at 26 °C and 200 rpm, the culture was centrifuged, and the cell pellet was recovered in M9 minimal medium containing 30 g/L of glucose. The experiments were performed in triplicate and maintained at 26 °C and 200 rpm for 120 h.

Production experiments were also performed using TB and M9-modified media. Overnight grown cultures of the best producer were used to inoculate 50 mL of each medium containing the antibiotics and an initial concentration of 4 g/L of glucose. The procedure was performed as described for LB+M9. However, 30 g/L of glucose was supplemented to this medium instead of changing the medium 5 h after the induction of protein expression. The experiments were performed in triplicate and maintained at 26 °C and 200 rpm for 120 h.

Samples (1 mL) were collected at specific time points for metabolite analysis. When TB and M9-modified media were used, an additional sample was collected to measure culture growth (OD_600nm_).

#### 3.5.3 Bioreactor Experiments

The best producing strain was grown overnight and then used to inoculate 100 mL of TB medium in a 500 mL shake flask. This culture was grown overnight at 37 °C and 200 rpm. Afterward, OD_600nm_ was measured, and the amount of culture to start bioreactor experiments at an OD_600nm_ of 0.1 was calculated. This volume of culture was centrifuged (5000 rpm, 10 min), and the cell pellet was resuspended in TB medium and used to inoculate the bioreactors. These experiments were conducted in duplicate in the 2L DASGIP Parallel Bioreactor System (Eppendorf).

Bioreactors were autoclaved at 121 °C for 20 min in 350 mL of TB medium. The other 50 mL of TB medium, to complete the 400 mL of working volume, was autoclaved in a shake flask to be used to resuspend the cell pellet to inoculate the bioreactor. The required antibiotics and 4 g/L of glucose were supplemented at the beginning of the experiment. An initial temperature of 37 °C, an agitation of 300 rpm, and a constant oxygen feeding of 0.5 vvm (12 L/h) were programmed. Stirring speed was adjusted to up to 1000 rpm to maintain a dissolved oxygen percentage (%DO) above 30%. pH control was done by feeding a 2 M NaOH solution to maintain a constant pH of 6.5. When the cultures reached an OD_600nm_ of 0.9, 0.1 mM IPTG was supplemented to the bioreactor to induce protein expression, and the temperature was changed to 26 °C. After 5 h, 30 g/L of glucose was supplemented, and the experiment was maintained for 120 h.

Another experiment was conducted with two additional pulses of glucose (10 g/L) supplemented at 24 and 48 h time points. These time points were chosen when the glucose concentration was around 5 g/L. Glucose consumption was evaluated using high-performance liquid chromatography (HPLC). The experiment was conducted for 144 h.

Samples (2 mL) were collected at specific time points for metabolite analysis and to measure OD_600nm_.

### 3.6 Metabolites Extraction and Analysis

After 96-deep well plate experiments, metabolites were extracted from 300 μL of whole cell broth using 100% (v/v) methanol in a 1:1 ratio. After vortexing to disrupt the cells and centrifugation (4000 rpm, 10 min), the supernatant was retrieved for UHPLC analysis. After shake flask and bioreactor experiments, metabolites were extracted from 1 mL of whole cell broth using 100% (v/v) ethyl acetate in a 1:1 ratio. Samples extraction, evaporation, and preparation for further UHPLC analysis were performed as described in Gomes et al.^17^

All of the extracted metabolites were analyzed by UHPLC using a Shimadzu Nexera-X2 system (Shimadzu Corporation, Kyoto, Japan). Kinetex 2.6 μm Polar C18 100 Å LC column (150 mm × 4.6 mm) (Phenomenex) was used in the analysis. Mobile phase A was composed of 0.1% (v/v) formic acid in water, and mobile phase B was composed of acetonitrile. A gradient of both mobile phases over time was used: 1–10 min, 5%–95% of B; 10–12 min, 95%–5% of B; and 12–15 min, 5% of B. SPD-M20A detector was programmed to measure the absorbance at 310 nm to detect *p-*coumaric acid and at 370 nm to detect naringenin chalcone, desmethylxanthohumol, and xanthohumol. *p-*Coumaric acid, naringenin chalcone, desmethylxanthohumol, and xanthohumol were quantified based on the peak areas of reference standards at 7.5, 9.0, 10.9, and 11.7 min.

The final bioreactor sample was also analyzed by LC–MS. Detection and identification of the compounds were performed using a Thermo Scientific Vanquish Flex UHPLC system coupled to a Thermo Scientific Orbitrap Exploris 120 high-resolution accurate mass spectrometer (Thermo Fisher Scientific, Bremen, Germany). The instrument control and data processing were carried out by Xcalibur 4.5 software (Thermo Fisher Scientific). The HPLC column utilized was a Kinetex C18 column (150 mm × 4.6 mm i.d., 2.6 μm particle size) protected with a guard column of the same material. The mobile phases consisted of A (water with 0.1% formic acid) and B (acetonitrile). The flow rate was 0.5 mL/min using the following gradient scheme (t in min; % B): 0–1 min, 5%; 1–10 min, 5–95%; 10–12 min, 95–5%; 12–15 min, 5%. The column temperature was 25 °C, the autosampler temperature was set at 15 °C, and the injection volume was 10 μL. Ion source (Thermo Scientific OptaMax NG ion source) was equipped with a heated electrospray ionization (HESI) probe. The external mass calibration of the Q-Orbitrap was performed once a week to ensure a working mass accuracy <3 ppm. The Orbitrap Exploris 120 mass spectrometer was equipped with a Heated Electrospray Ionization (HESI) source. The optimized HESI temperature was set at 350 °C, the capillary temperature at 350 °C, the electrospray voltage at 3.5 kV and 2.5 kV for positive and negative modes, respectively. Sheath and auxiliary gas were 60 and 15, respectively. All qualitative data in this study were acquired using SIM and MS^2^. In MS^2^, if the targeted compounds were detected, precursor ions that were selected by the quadrupole were sent to the HCD collision cell of the Q-Orbitrap mass spectrometer. Here, they were fragmented with an NCE to obtain product ion spectra. Target mass list for MS^2^ was performed with a resolution of 15,000 and NCE 30%.

Glucose consumption was evaluated in shake flask and bioreactor experiments using a JASCO HPLC system coupled with an RI-203 detector. Aminex HPX-87 H column (Bio-Rad) was kept at 60 °C, and 5 mM H_2_SO_4_ was used as mobile phase at a constant flow rate of 0.5 mL/min. Glucose was quantified based on the peak area of reference standards at 10.9 min.

### 3.7 Statistical Analysis

Statistical analysis was performed using GraphPad Prism Software, Inc., version 8.0.1. Statistical significance was concluded when the *p-*value was <0.05 using Tukey’s multiple comparisons test for comparison of more than two conditions and the unpaired *t*-test with Welch’s correction for the comparison between only two conditions. The results of 96-deep well plate and flask experiments are presented as the mean value of three independent tests ± the standard deviation. The results of bioreactor experiments are presented as the mean value of two independent tests ± the standard deviation.

## Supplementary Material



## Notes

Strains, plasmids, and all of the primers used in this study; sequence of *Hl*OMT1 and *metK*; SDS-PAGE gel showing HlOMT1 expression, experiment reporting the production of naringenin chalcone by *E. coli* M-PAR-121 expressing pRSFDuet_*Fj*TAL_*Cm*CHS_*At*4CL; agarose gel to confirm the correct integration of *S-*adenosylmethionine (SAM) synthase (*metK*) in the several DMAPP-*E. coli* M-PAR-121-boosted strains; representative chromatograms of standard and bioreactor experiment sample, and characterization and fragmentation spectra of the standards and produced compounds obtained in the LC-MS analysis (PDF)

## Abbreviations

4CL: 4-coumarate-CoA ligase 1
*ahpC*: alkyl hydroperoxide reductase C
CA: *p-*coumaric acid
CHS: chalcone synthase
CRISPR-Cas12a: clustered regularly interspaced short palindromic repeats (CRISPR) and CRISPR-associated protein 12a (Cas12a)
DMAPP: dimethylallyl pyrophosphate
DMX: desmethylxanthohumol
DXS: 1-deoxy-d-xylulose-5-phosphate synthase
FPP: farnesyl diphosphate
IDI: isopentenyl diphosphate isomerase
*lacZ*: β-galactosidase
*metK*: SAM synthase
NC: naringenin chalcone
OD_600 nm_: optical density at 600 nm
OMT: *O-*methyltransferase
PT: prenyltransferase
SAM: *S-*adenosylmethionine
SDS-PAGE: sodium dodecyl sulfate (SDS) polyacrylamide gel electrophoresis (SDS-PAGE)
TAL: tyrosine-ammonia lyase
TB: terrific broth
XAN: xanthohumol
