# Supplementary material for: De Novo Production of Xanthohumol by a Metabolically Engineered Escherichia coli
Source: ACS Synth Biol. 2025 Sep 25;14(10):3913–26. doi: 10.1021/acssynbio.5c00221 (PMC12818835; doi:10.1021/acssynbio.5c00221)
Supplement: Supplementary file 1 [file sb5c00221_si_001.pdf]

Supplementary material

De novo* production of xanthohumol by a metabolically engineered *Escherichia coli

Daniela Gomes,[1] Joana Santos,[1] Armando Venâncio,[1,2] Joana L. Rodrigues,[1,2*]
Nigel S. Scrutton,[3] Ligia R. Rodrigues [1,2]

[1] CEB-Centre of Biological Engineering, Universidade do Minho, Campus de Gualtar, 4710-057, Braga, Portugal

[2] LABBELS- Associate Laboratory, Braga/Guimarães

[3] Manchester Institute of Biotechnology, The University of Manchester, 131 Princess Street, Manchester M1 7DN, UK

* Corresponding author: joanarodrigues@deb.uminho.pt; Phone: +351 253 604402

Table S1. Strains used in this study.

Strains	Relevant Genotype	Source
E. coli NEB5α	*fhuA2Δ(argF-lacZ)*U169 *phoA glnV44 Φ80Δ(lacZ)M15 gyrA96 recA1 relA1 endA1 thi-1 hsdR17*	(New England Biolabs - C2987H)
E. coli NZY5	*fhuA2Δ(argF-lacZ)*U169 *phoA glnV44* Φ80 *Δ(lacZ)*M15 *gyrA96 recA1 relA1 endA1 thi-1 hsdR17*	NZYTech (MB00401)
E. coli M-PAR-121	*L*-Tyrosine overproducing strain derived from MG1655 (DE3); *tyrR*::P_{T7lac}-$aroG^{fbr}$ *ldhA*::P_{T7lac}-$tyrA^{fbr}$ *adhE*::P_{T7lac}-*ppsA pflDC*::$P_{T7lac(-8TC)}$-*tktA pykF*::P_{T7lac}-*aroALC ascF*::P_{T7lac}-*aroEDB*	[1]
E. coli M-PAR-121:*Bs*DXS	*E. coli* M-PAR-121 with the integration of 1-deoxy-D-xylulose-5-phosphate synthase (DXS) gene from *Bacillus subtilis* (*Bs*DXS) into the *lacZ* locus of the genome	[2]
E. coli M-PAR-121:*Sc*IDI	*E. coli* M-PAR-121 with the integration of isopentenyl diphosphate isomerase (IDI) from *Saccharomyces cerevisiae* (*Sc*IDI) into the *lacZ* locus of the genome	[2]
E. coli M-PAR-121:*Bl*IDI	*E. coli* M-PAR-121 with the integration of IDI from *Bacillus licheniformis* (*Bl*IDI) into the *lacZ* locus of the genome	[2]
E. coli M-PAR-121:*Bs*DXS-*Sc*IDI	*E. coli* M-PAR-121 with the integration of *Bs*DXS and *Sc*IDI into the *lacZ* locus of the genome	[2]
E. coli M-PAR-121:*Bs*DXS-*Bl*IDI	*E. coli* M-PAR-121 with the integration of *Bs*DXS and *Bll*IDI into the *lacZ* locus of the genome	[2]
E. coli M-PAR-121:*Ec*DXS	*E. coli* M-PAR-121 with the integration of DXS gene from *Escherichia coli* (*Ec*DXS) into the *lacZ* locus of the genome	[2]
E. coli M-PAR-121:*Ec*IDI	*E. coli* M-PAR-121 with the integration of IDI gene from *E. coli* (*Ec*IDI) into the *lacZ* locus of the genome	[2]
E. coli M-PAR-121:*Ec*DXS-*Ec*IDI	*E. coli* M-PAR-121 with the integration of *Ec*DXS and *Ec*IDI into the *lacZ* locus of the genome	[2]
E. coli M-PAR-121:*metK*	*E. coli* M-PAR-121 with the integration of *S*-adenosylmethionine (SAM) synthase (*metK*) into the *ahpC* locus of the genome, respectively	This study
E. coli M-PAR-121:*Bs*DXS:*metK*	*E. coli* M-PAR-121 with the integration of *Bs*DXS and *metK* into the *lacZ* locus and *ahpC* locus of the genome, respectively	This study
E. coli M-PAR-121:*Sc*IDI:*metK*	*E. coli* M-PAR-121 with the integration of *Sc*IDI and *metK* into the *lacZ* locus and *ahpC* locus of the genome, respectively	This study
E. coli M-PAR-121:*Bl*IDI:*metK*	*E. coli* M-PAR-121 with the integration of *Bl*IDI and *metK* into the *lacZ* locus of the genome and *ahpC* locus of the genome, respectively	This study
E. coli M-PAR-121:*Bs*DXS-*Sc*IDI:*metK*	*E. coli* M-PAR-121 with the integration of *Bs*DXS and *Sc*IDI into the *lacZ* locus of the genome and *metK* into the *ahpC* locus of the genome	This study
E. coli M-PAR-121:*Bs*DXS-*Bl*IDI:*metK*	*E. coli* M-PAR-121 with the integration of *Bs*DXS and *Bll*IDI into the *lacZ* locus of the genome and *metK* into the *ahpC* locus of the genome	This study
E. coli M-PAR-121:*Ec*DXS:*metK*	*E. coli* M-PAR-121 with the integration of *Ec*DXS and *metK* into the *lacZ* locus of the genome and *ahpC* locus of the genome, respectively	This study
E. coli M-PAR-121:*Ec*IDI:*metK*	*E. coli* M-PAR-121 with the integration of *Ec*IDI and *metK* into the *lacZ* locus of the genome and *ahpC* locus of the genome, respectively	This study
E. coli M-PAR-121:*Ec*DXS-*Ec*IDI:*metK*	*E. coli* M-PAR-121 with the integration of *Ec*DXS and *Ec*IDI into the *lacZ* locus of the genome and *metK* into the *ahpC* locus of the genome	This study

Table 5.2. Plasmids used in this study.

Plasmids	Construct	Source
pCDFDuet-1	CloDF13 ori, lacI, double PT7lac, $Spec^R$	Novagen
pRSFDuet_*Fj*TAL_*Cm*CHS	pRSFDuet-1 (RSF1030 ori, lacI, double PT7lac, Kan^R) carrying codon-optimized tyrosine-ammonia lyase (TAL) from *Flavobacterium johnsoniae (Fj*TAL*)* and chalcone synthase (CHS) from *Curcubita maxima* (*Cm*CHS)	3
pACYCDuet_*At*4CL	pACYCDuet-1 (P15A ori, lacI, double PT7lac, Cm^R) carrying 4-coumarate-CoA ligase 1 (4CL-1) from *Arabidopsis thaliana* (*At*4CL)	4
pSIM*cpf1*	pSIM18 derived vector, Rep101 ori, Hyg^R, P_{BAD}-α-pMB1 array, $P_{JS23151}$ *Ascpf1*	5
pTF-*ahpC-rfp*	pMB1 ori, $Spec^R$, $P_{JS23119}$-*lacZ* array, *ahpC*::*rfp*	5
pCDFDuet_*Hl*PT1	pCDFDuet-1 carrying codon-optimized PT1 from *Humulus lupulus* (*Hl*PT1)	2
pCDFDuet_*Sf*N8DT-1	pCDFDuet-1 carrying codon-optimized N8DT-1 from *Sophora flavescens* (*Sf*N8DT-1)	2
pCDFDuet_AnaPT	pCDFDuet-1 carrying prenyltransferase (PT) AnaPT from *Neosartorya fischeri*	2
pCDFDuet_CdpC3PT	pCDFDuet-1 carrying CdpC3PT from *N. fischeri*	2
pCDFDuet_*Cs*PT3	pCDFDuet-1 carrying a codon-optimized version of PT3 from *Cannabis sativa* (*Cs*PT3)	2
pCDFDuet_CoAnaPT	pCDFDuet-1 carrying a codon-optimized version of AnaPT from *N. fischeri* (coAnaPT)	2
pCDFDuet_CloQ	pCDFDuet-1 carrying a codon-optimized version of CloQ from *Streptomyces roseochromogenes*	2
pCDFDuet_*Ec*PT	pCDFDuet-1 carrying a codon-optimized version of PT from *E. coli* (*Ec*PT)	2
pCDFDuet_NphB	pCDFDuet-1 carrying a codon-optimized version of NphB from *Streptomyces* sp.	2
pCDFDuet_*Sp*PT	pCDFDuet-1 carrying a codon-optimized version of PT from *Streptomyces* sp. Act143 (*Sp*PT)	2
pCDFDuet_UbiA	pCDFDuet-1 carrying a codon-optimized version of UbiA from *E. coli*	2
pRSFDuet_*Fj*TAL_*Cm*CHS_*At*4CL	pRSFDuet-1 (RSF1030 ori, lacI, double PT7lac, Kan^R) carrying *Fj*TAL*, At*4CL, and *Cm*CHS	This study
pTF-*ahpC-metK*	pMB1 ori, $Spec^R$, $P_{JS23119}$-*lacZ* array, *ahpC*::*metK*	This study
pCDFDuet_*Hl*OMT1	pCDFDuet-1 carrying codon-optimized *O*-methyltransferase 1 (OMT1) from *H. lupulus* (*Hl*OMT1)	This study
pCDFDuet_*Hl*PT1_*Hl*OMT1	pCDFDuet-1 carrying *Hl*PT1 and *Hl*OMT1	This study
pCDFDuet_*Sf*N8DT-1_*Hl*OMT1	pCDFDuet-1 carrying *Sf*N8DT-1 and *Hl*OMT1	This study
pCDFDuet_AnaPT_*Hl*OMT1	pCDFDuet-1 carrying AnaPT *Hl*OMT1	This study
pCDFDuet_CdpC3PT_*Hl*OMT1	pCDFDuet-1 carrying CdpC3PT and *Hl*OMT1	This study
pCDFDuet_*Cs*PT3_*Hl*OMT1	pCDFDuet-1 carrying carrying *Cs*PT3 and *Hl*OMT1	This study
pCDFDuet_CoAnaPT_*Hl*OMT1	pCDFDuet-1 carrying a coAnaPT and *Hl*OMT1	This study
pCDFDuet_CloQ_*Hl*OMT1	pCDFDuet-1 carrying CloQ and *Hl*OMT1	This study
pCDFDuet_*Ec*PT_*Hl*OMT1	pCDFDuet-1 carrying *Ec*PT and *Hl*OMT1	This study
pCDFDuet_NphB_*Hl*OMT1	pCDFDuet-1 carrying NphB and *Hl*OMT1	This study
pCDFDuet_*Sp*PT_*Hl*OMT1	pCDFDuet-1 carrying *Sp*PT and *Hl*OMT1	This study
pCDFDuet_UbiA_*Hl*OMT1	pCDFDuet-1 carrying UbiA and *Hl*OMT1	This study

Table S3. Sequence of the tested *O*-methyltransferase 1 (OMT1) from *Humulus lupulus* (*Hl*OMT1).

Gene	Sequence
***Hl*OMT1**	atggagtccttacgcgggcaggagcaaatctggcagttaatgttttccttcgtggatagtatggcgctcaagtgtgcaattgaatta<br>cgtatcgcagatattatacatagccacggaaagccaattacactttctcagattgccagtgggattcggagcaattctaattcatcga<br>tttcaccaaacataccatatctgtcgcgtattatgcgtttcttagtacgcaagaacatatttacggagcaccaagaggacaacgacg<br>aagttatttctctctatggcctttcggactcttctcgttggcttctccgtgacttcaaatcttctcttgcgccgatggtcttaatgcaaacc<br>cacccgcttagtatggcagtctggcactttctggaagactacgttcgaaattcctcaaatacctttgagaaagcccatggatgcaat<br>atctgggaattttcgagcgcgaaccccgagtttaataaaatctttaataacgcgatggcctcgatagtcccgatttatatgggagcc<br>atgttaagcagctacaaagacggcctgggatgcataaagggcactgtcgtcgatgtgggtggcgggacaggcggaagcatctc<br>cgaactgatgaagtactaccctaatattaagggtataaatttcgatttgccgcacgtcatcgctacggcccctgcccttgacggagt<br>gacgcacatctctggagacatctttgaaagcatcccatctgcggacgcagtgctgatgaaaggcgtccttcactgcttttcagacg<br>agaagtgcgttaaggttctccgaaactgccgcaaggccattaccgataagaagaacggtaaaataattatccttgaaatagtcctg<br>gaccccacgagtaaccagattttcgatgaaacccgcatggtctatgacctgttaatacctctgttctcgggcggcaaggaacgtac<br>agaactggagtggaagcgtttacttaatgaagcggggttcacgtgcattaagattactaagataccgatcattccggccatcatcg<br>aagcgttcctggtttaa

Table S4. List of primers used for the construction and verification of pathway plasmids.

Primer	Sequence[1]
***At*4CL-insert-Fwd**	**AACGTCGTCGCTAAC**GCGACTCCTGCATTAGGAAA
***At*4CL-insert-Rev**	**CTTTACCAGACTCGATCAG**TCACAATCCATTTGCTAGTTTT
**pRSFDuet-*Fj*TAL-*Cm*CHS-Fwd**	TCGAGTCTGGTAAAGAAACCGC
**pRSFDuet-*Fj*TAL-*Cm*CHS-Rev**	GTTAGCGACGACGTTTCGGC
***Hl*OMT1-Fwd**	**GAAGGAGATATACAT*ATG***GAG
***Hl*OMT1-Rev**	**ATTGAGATCTGCCAT*TTA***AACCAGGAACGCT
**pCDFDuet-linearMCS2-Fwd**	ATGGCAGATCTCAATTG
**pCDFDuet-linearMCS2-Rev**	ATGTATATCTCCTTCTTATACTTA
***Cm*CHS-sequencing-Fwd**	CCGAAACGTCGTCGCTAA
**MCS2-Fwd**	GTACACGGCCGCATAATCG
**T7terminator-Rev**	CTAGTTATTGCTCAGCGGT

[1] Start and stop codons in *italic*;; homology arms in **bold**.

Table S5. Sequence of the *metK* gene used in the genome integration strategies.

Gene	Sequence
metK	atggcaaaacacctttttacgtccgagtccgtctctgaagggcatcctgacaaaattgctgaccaaatttctgatgccgttttagacg<br>cgatcctcgaacaggatccgaaagcacgcgttgcttgcgaaacctacgtaaaaaccggcatggttttagttggcggcgaaatca<br>ccaccagcgcctgggtagacatcgaagagatcacccgtaacaccgttcgcgaaattggctatgtgcattccgacatgggctttga<br>cgctaactcctgtgcggttctgagcgctatcggcaaacagtctcctgacatcaaccagggcgttgaccgtgccgatccgctggaa<br>cagggcgcgggtgaccagggtctgatgtttggctacgcaactaatgaaaccgacgtgctgatgccagcacctatcacctatgca<br>caccgtctggtacagcgtcaggctgaagtgcgtaaaaacggcactctgccgtggctgcgcccggacgcgaaaagccaggtga<br>cttttcagtatgacgacggcaaaatcgttggtatcgatgctgtcgtgctttccactcagcactctgaagagatcgaccagaaatcgc<br>tgcaagaagcggtaatggaagagatcatcaagccaattctgcccgctgaatggctgacttctgccaccaaattcttcatcaacccg<br>accggtcgtttcgttatcggtggcccaatgggtgactgcggtctgactggtcgtaaaattatcgttgatacctacggcggcatggc<br>gcgtcacggtggcggtgcattctctggtaaagatccatcaaaagtggaccgttccgcagcctacgcagcacgttatgtcgcgaaa<br>aacatcgttgctgctggcctggccgatcgttgtgaaattcaggtttcctacgcaatcggcgtggctgaaccgacctccatcatggta<br>gaaactttcggtactgagaaagtgccttctgaacaactgaccctgctggtacgtgagttcttcgacctgcgcccatacggtctgatt<br>cagatgctggatctgctgcacccgatctacaaagaaaccgcagcatacggtcactttggtcgtgaacatttcccgtgggaaaaaa<br>ccgacaaagcgcagctgctgcgcgatgctgccggtctgaagtaa

Table S6. List of primers used for the construction and verification of CRISPR-Cas12a plasmids.

Primer	Sequence[1]
**pTF-integration-*ahpC*-Fwd**	ATTTCCTTCGTCTTTCACGCC
**pTF-integration-*ahpC*-Rev**	GTCTACGCCCAGTTTCTGC
***metK*-integration-Fwd**	**AAACTGGGCGTAGAC***ATG*GCAAAACACCTTTTTAC
***metK*-integration-Rev**	**AAAGACGAAGGAAAT***TTA*CTTCAGACCGGCAG
**pTF-*ahpC*-sequencing-Fwd**	GAACTGCAGAAACTGGGCGT
**pTF-*ahpC*-sequencing-Rev**	CGACGCCAACGCCGCTAT

[1] Start and stop codons in *italic*; homology arms in **bold**.

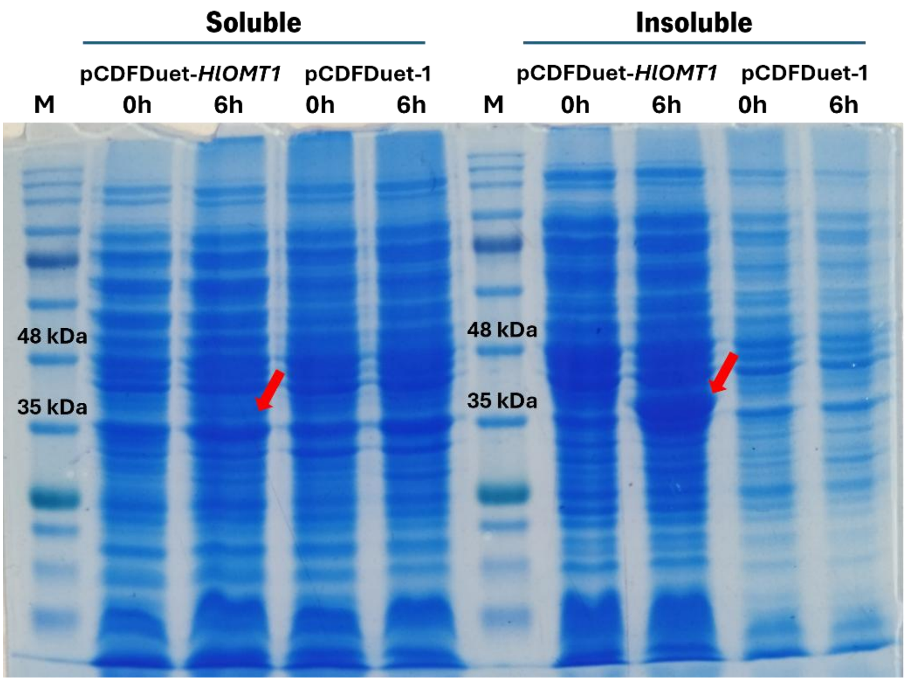

Figure S1. Protein sodium dodecyl sulfate (SDS) polyacrylamide gel electrophoresis (SDS-PAGE) gel of soluble and insoluble protein fractions showing *O*-methyltransferase 1 (OMT1) from *Humulus lupulus* (*Hl*OMT1) expression in *E. coli* M-PAR-121 strain at time zero (0 h) of induction and after 6 h of induction. Protein band is expected to be around 39.18 kDa. *E. coli* M-PAR-121 carrying pCDFDuet-1 was used as a control strain. NZYColour Protein Marker II (NZYTech) was used as protein reference standard.

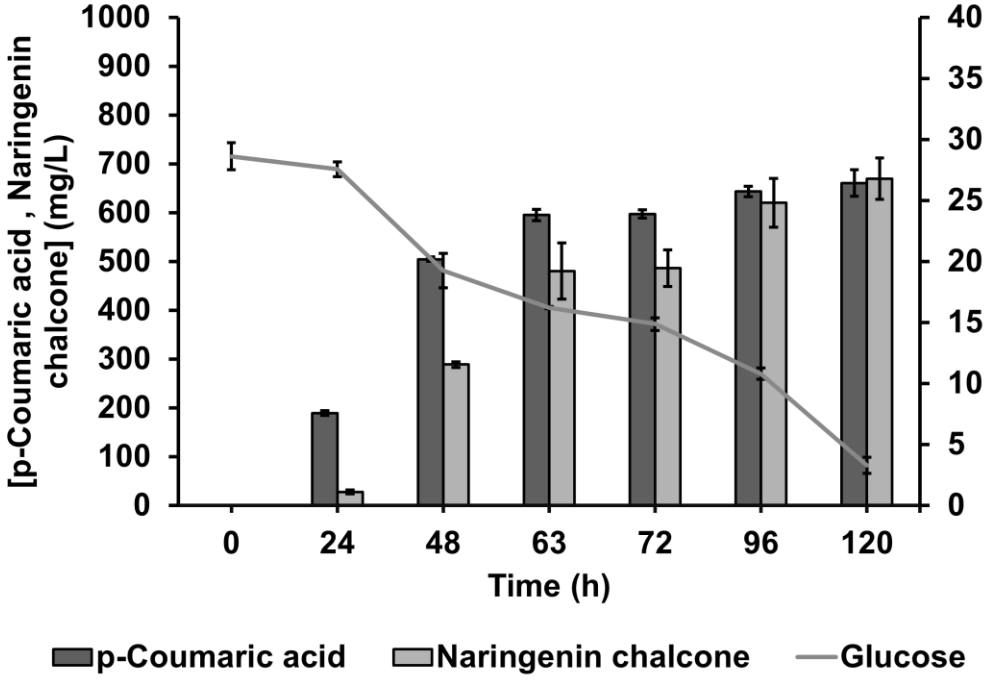

Figure S2. Profile of glucose consumption and metabolites production by *Escherichia coli* M-PAR-121 expressing pRSFDuet_*Fj*TAL_*Cm*CHS_*At*4CL in shake flask experiments using the combination of LB+M9. Results correspond to the average of three independent experiments ± standard deviation.

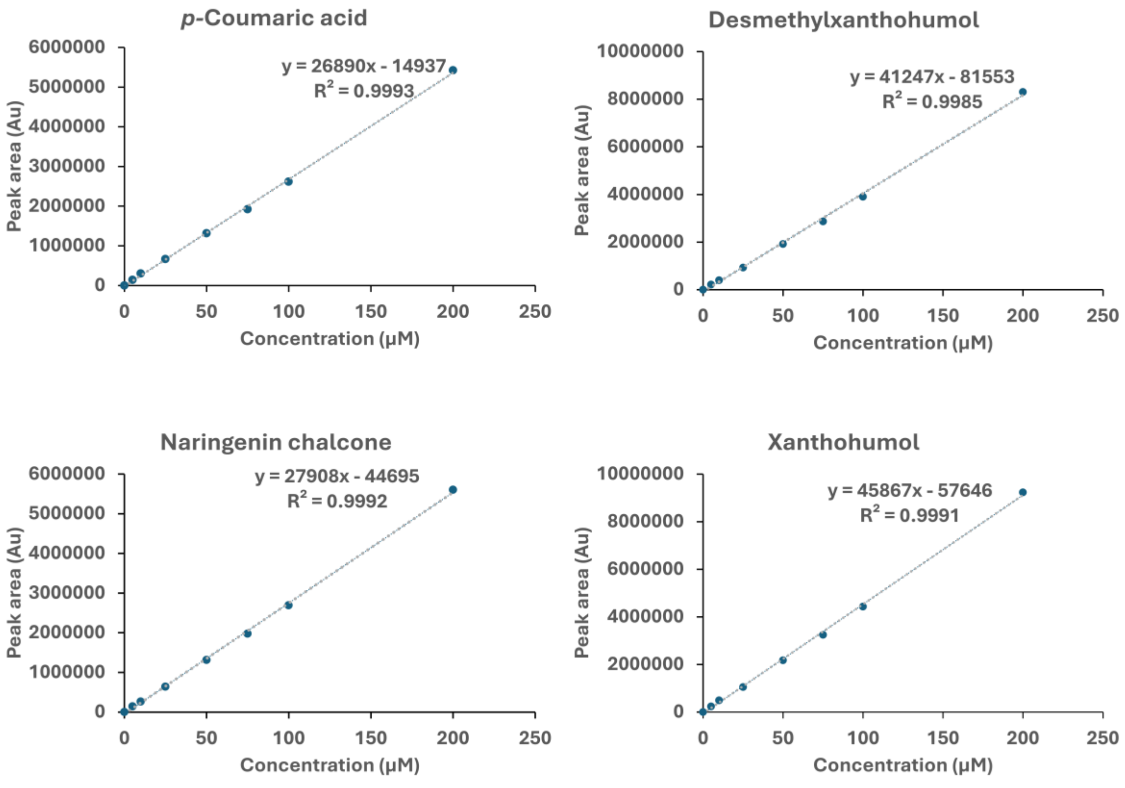

Figure S3. Calibration plots for the analytical standards *p*-coumaric acid, naringenin chalcone, desmethylxanthohumol and xanthohumol.

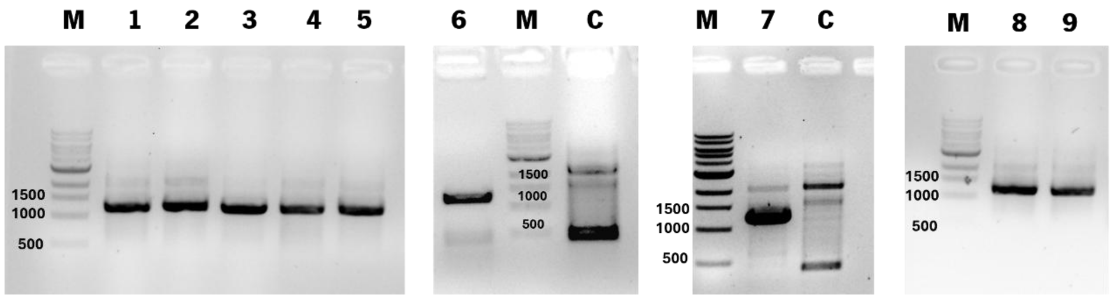

Figure S4. Agarose gel 1% (w/v) to confirm the correct integration of *S*-adenosylmethionine (SAM) synthase (*metK*) into the *ahpC* locus of *E. coli* M-PAR-121 and dimethylallyl diphosphate (DMAPP) modified *E. coli* M-PAR-121 strains. Lane 1: integration into *E. coli* M-PAR-121 (wild-type). Lane 2: integration into *E. coli* M-PAR-121:*Bs*DXS. Lane 3: integration into *E. coli* M-PAR-121:*Sc*IDI. Lane 4: integration into *E. coli* M-PAR-121:*Bs*DXS-*Sc*IDI. Lane 5: integration into *E. coli* M-PAR-121:*Bs*DXS-*Bl*IDI. Lane 6: integration into *E. coli* M-PAR-121:*Bl*IDI. Lane 7: integration into *E. coli* M-PAR-121:*Ec*DXS. Lane 8:integration into *E. coli* M-PAR-121:*Ec*IDI. Lane 9: integration into *E. coli* M-PAR-121: *Ec*DXS-*Ec*IDI. The expected band size for the successful integration of *metK* is 1218 bp. Lane C corresponds to the control PCR performed with genomic DNA of the wild-type strain. The expected size for this fragment is 425 bp. Unspecific products are displayed in the control. Lane M corresponds to 1 kb DNA ladder (NEB) and the sizes of the bands of interest are represented in bp.

Reference standard

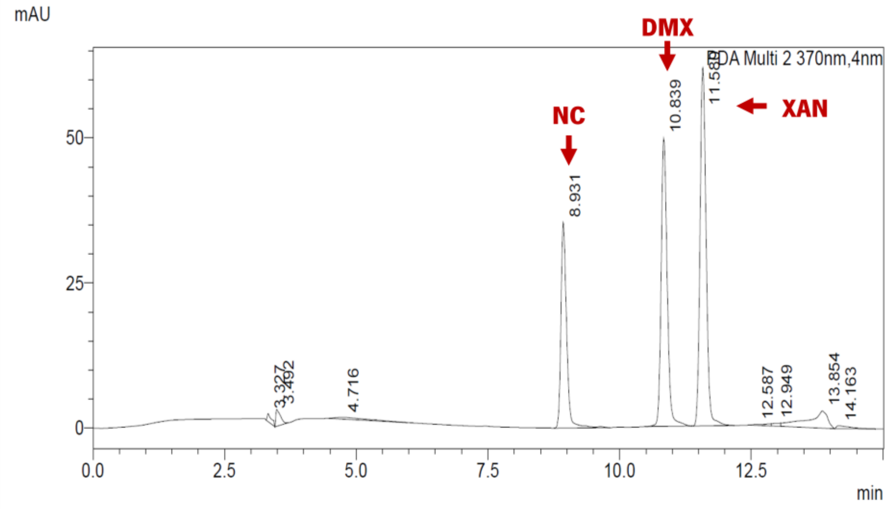

Experiment Sample at 370 nm

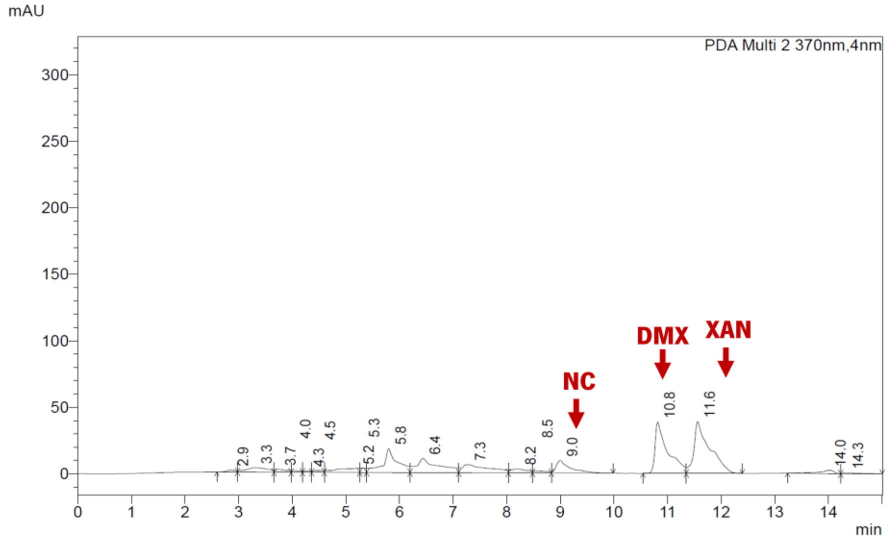

Figure S5. Representative chromatograms at 370 nm of the analytical standard (25 µM) composed by naringenin chalcone (NC), desmethylxanthohumol (DMX), and xanthohumol (XAN) and one sample from the bioreactor experiment with higher production levels. *p*-coumaric acid (CA) (retention time at 7.5 min) is present in the sample and standard but it is only detected and quantified at 310 nm.

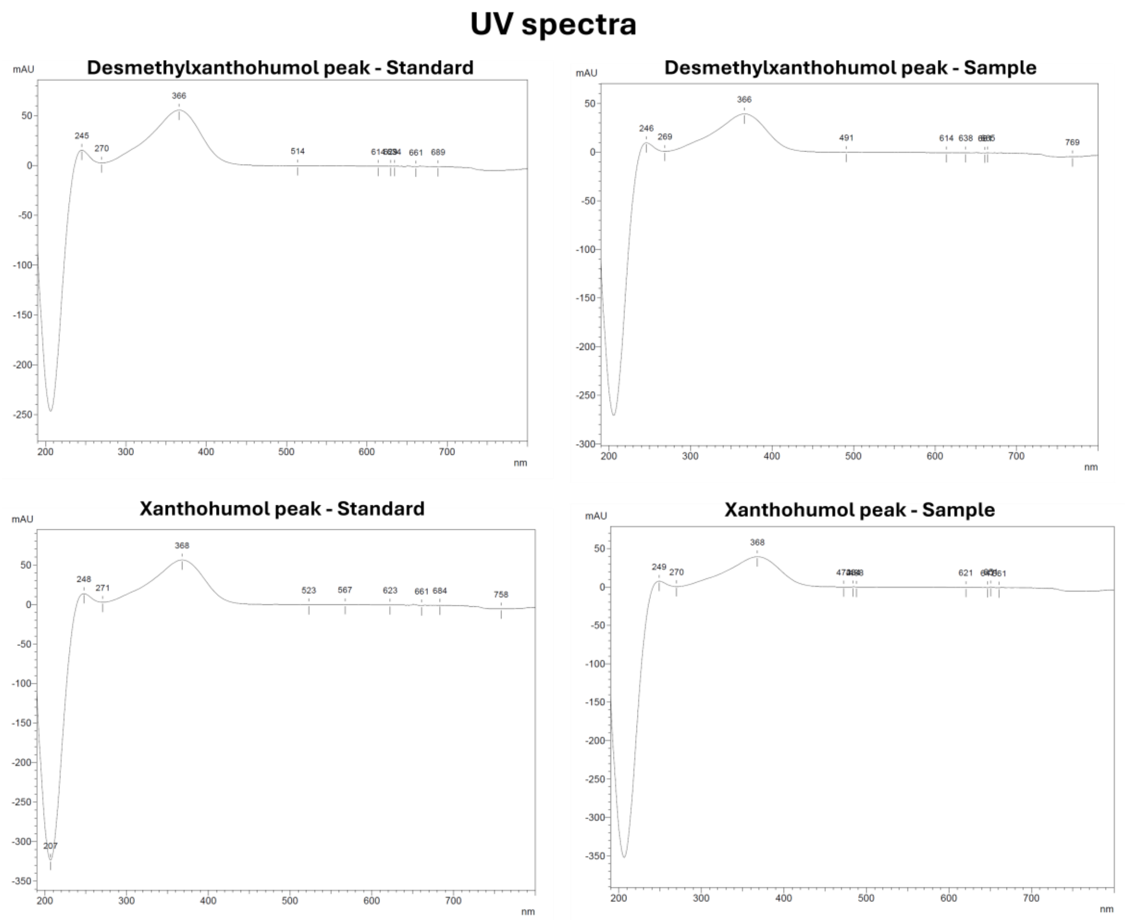

Figure S6. Comparison of the UV spectra of desmethylxanthohumol and xanthohumol peaks from analytical standards (25 µM) (left) with the corresponding peaks from a bioreactor sample exhibiting the highest production levels (right).

Table S7 – Characterization of the standard compounds by mass spectrometry.

	Compound	RT [min]	Chemical formula	Exact Mass	Accurate Mass $(M-H)^-$	Experimental Adduct Ion *(m/z)*
**1**	Desmethylxanthohumol	12.07	$C_{20}H_{20}O_5$	340.131	339.123	339.123
**2**	Xanthohumol	12.78	$C_{21}H_{22}O_5$	354.146	353.139	353.139

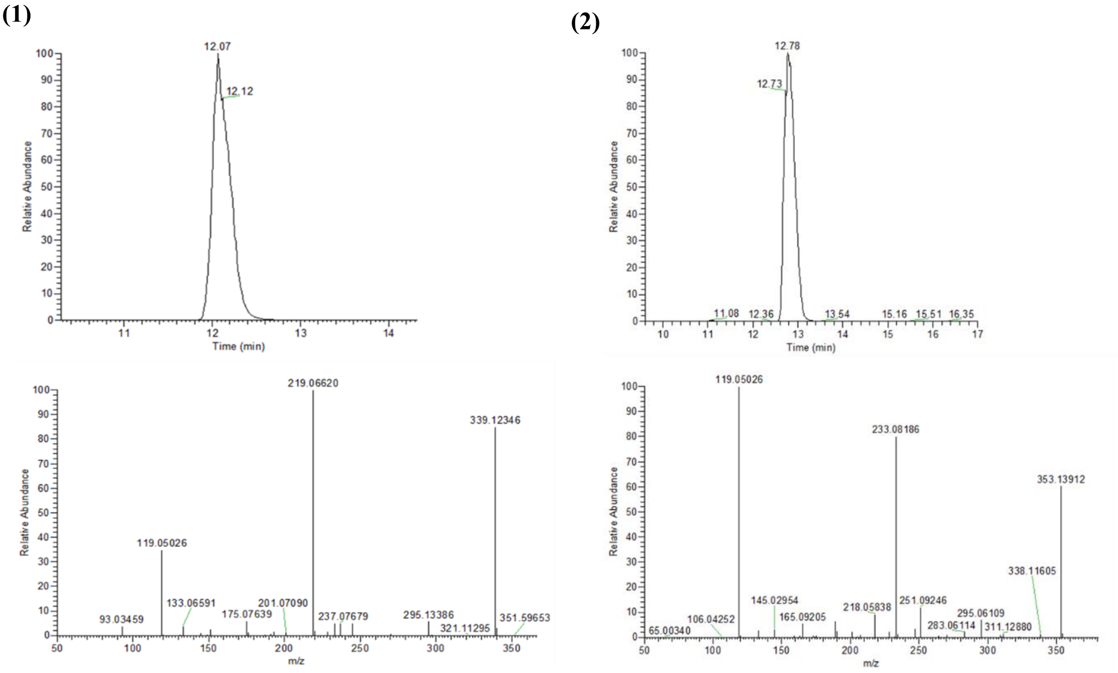

Figure S7. Chromatogram and fragmentation spectra (MS^2) obtained for (1) Desmethylxanthohumol and (2) Xanthohumol from standard solutions.

(1) (2)

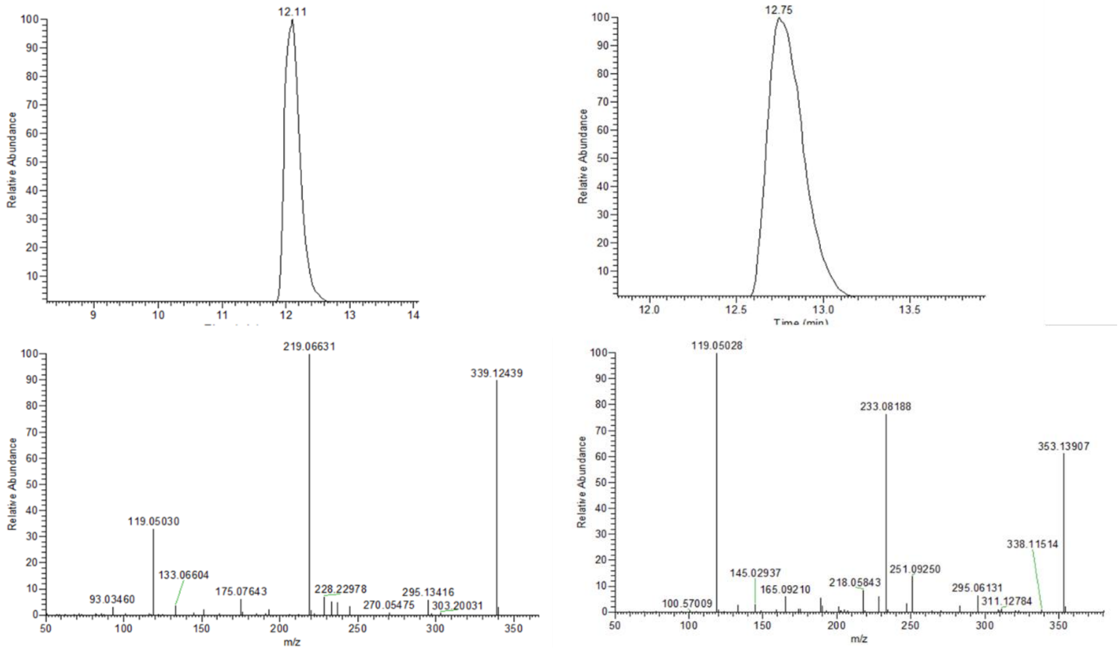

Figure S8. Chromatogram and fragmentation spectra (MS^2) obtained from the sample analysis, corresponding to the precursor ions *m/z* 339.123 (1) and 353.139 (2).

Table S8.- Characteristic fragments of each compound from the sample analysis found in the MS^2 spectrum (30% NCE).

	**Molecular ion (M-H)$^+$**	**Fragment 1**	**Fragment 2**	**Fragment 3**	**Fragment 4**	**Fragment 5**	**Fragment 6**
**1**	339.123	295.134	219.066	175.076	133.066	119.050	93.034
**2**	353.139	295.061	251.092	233.081	218.058	165.092	119.050

References

(1) Koma, D.; Kishida, T.; Yoshida, E.; Ohashi, H.; Yamanaka, H.; Moriyoshi, K.; Nagamori, E.; Ohmoto, T. Chromosome Engineering to Generate Plasmid-Free Phenylalanine- and Tyrosine-Overproducing *Escherichia coli* Strains That Can Be Applied in the Generation of Aromatic-Compound-Producing Bacteria. *Appl Environ Microbiol* 2020, *86* (14), 1–24. https://doi.org/10.1128/AEM.00525-20.

(2) Gomes, D.; Rodrigues, J. L.; Scrutton, N. S.; Rodrigues, L. R. *De novo* Production of Prenylnaringenin Compounds by a Metabolically Engineered *Escherichia coli*. *bioRxiv* 2025, 1–37. (*Preprint*) https://doi.org/10.1101/2025.05.09.653005.

(3) Gomes, D.; Rodrigues, J. L.; Rodrigues, L. R. Step-by-Step Optimization of a Heterologous Pathway for *de novo* Naringenin Production in *Escherichia coli*. *Appl Microbiol Biotechnol* 2024, *108* (1). https://doi.org/10.1007/s00253-024-13271-7.

(4) Rodrigues, J. L.; Gomes, D.; Rodrigues, L. R. A Combinatorial Approach to Optimize the Production of Curcuminoids from Tyrosine in *Escherichia coli*. *Front Bioeng Biotechnol* 2020, *8* (59), 1–15. https://doi.org/10.3389/fbioe.2020.00059.

(5) Jervis, A. J.; Hanko, E. K. R.; Dunstan, M. S.; Robinson, C. J.; Takano, E.; Scrutton, N. S. A Plasmid Toolset for CRISPR-Mediated Genome Editing and CRISPRi Gene Regulation in *Escherichia coli*. *Microb Biotechnol* 2021, *14* (3), 1120–1129. https://doi.org/10.1111/1751-7915.13780.
